# ABA-based teacher training reduces emotional and behavioral problems in Brazilian children

**DOI:** 10.1186/s41155-026-00385-2

**Published:** 2026-03-25

**Authors:** Rayra Santos de Souza, Patrícia Moraes Cabral, Carolina Kulcsar Caravieri, Maria Clara Souza Neder, Maria Cristina Triguero Veloz Teixeira

**Affiliations:** Human Developmental Sciences Graduate Program, Mackenzie Presbyterian University, Rua da Consolação, 930, Consolação, São Paulo, CEP 01302-907 SP Brasil

**Keywords:** Emotional and behavioral problems, Teacher, Behavioral training, School, Children

## Abstract

**Background:**

COVID-19 containment measures have led to an increase in emotional and behavioral problems (EBP) in children. Studies show positive effects of interventions with teacher training in reducing EBP in children.

**Objective:**

This study aimed to implement and verify the effects of an online ABA-based teacher training program on prosocial behaviors and EBP in students returning to in-person classes during the COVID-19 pandemic.

**Methods:**

Participants included 345 parents/caregivers of 2nd and 3rd grade elementary school students and their 37 teachers, divided into a control group (CG) and an intervention group (IG). Teachers in the IG received online ABA-based training focused on EBP and prosocial behaviors contents, while those in the CG were exposed to reading strategies contents. The Strengths and Difficulties Questionnaire (SDQ) (parents and teachers), sociodemographic and socioeconomic questionnaires, and checklists to monitor strategies used by teachers were used.

**Results:**

The results showed that parents in the IG perceived a decrease in EBP, with statistically significant results in conduct problems (p=0.046) and hyperactivity (p=0.039), while teachers perceived a reduction in hyperactivity (p=0.007) and emotional problems (p=0.043) in the post-intervention (Time 2). The greater the use of ABA-based strategies by teachers, the lower the occurrence of EBPs among students (r=-0.31). No significant reductions in EBP were observed in the CG.

**Conclusion:**

These results indicate that online ABA-based teacher training is a low-cost intervention that has produced positive effects in promoting students' mental health, including reducing EBP and improving students' prosocial behavior.

**Supplementary Information:**

The online version contains supplementary material available at 10.1186/s41155-026-00385-2.

## Introduction

Emotional and behavioral problems (EBP) in childhood and adolescence have been linked with lower academic performance (Mundy et al., 2017; Pagerols et al., 2022) and the development of psychiatric disorders such as depression and anxiety in adulthood (Akingbuwa et al., 2020; Copeland et al., 2009; Loth et al., 2014). EBP can be classified into: (a) internalizing problems, encompassing behaviors that predominantly affect the individual themselves, such as anxiety, withdrawal, somatic complaints with an emotional background and depression; and (b) externalizing problems, which predominantly affect other people, for example, aggressive behavior, rule breaking, and substance misuse (Achenbach & Edelbrock, 1978; Achenbach & Rescorla, 2001).

Previous studies conducted before the COVID-19 pandemic already showed high rates of EP in children (Bach et al., 2019; Kieling et al., 2011; Magai et al., 2018; Paula et al., 2007; Srinath et al., 2010; Teixeira et al., 2014). However, the context of the COVID-19 pandemic increased the occurrence of EBP in the child population and worsened it in those who already presented it pre-pandemic (Golberstein et al., 2020; Meherali et al., 2021; Panda et al., 2021; Ravens-Sieberer et al., 2022). Confinement in home environments with a greater number of family members occupying the same spaces, increase family conflicts and marital discord, lack of leisure in open spaces, restrictions on sports activities, remote classes, a significant decrease in social interactions, parental stress, parental psychiatric disorders, the increased complexity of family routines and excessive video game use are some examples of the negative factors that contributed to the increase in EBP in the child population due to social isolation (Garcia et al. 2020; Panda et al. 2021; Riter et al. 2021; Sesso et al. 2021; Souza et al. 2022; Wang et al. 2021a).

Studies have verified the occurrence of EBP in the child population during the pandemic with comparative data with the pre-pandemic period (Khoury et al., 2021; Ravens-Sieberer et al., 2022; Samji et al., 2021). For example, a longitudinal study by Zuccolo et al. (2023) assessed emotional problems in children and adolescents living in Brazil (*n* = 5795) during the pandemic through biweekly assessments (from June 2020 to June 2021). Comparing the measures collected at the beginning of the study with the final period, there was a significant increase in emotional problems and depressive symptoms; however, this increase was not sustained throughout the pandemic, with fluctuations identified that were associated with periods with greater social mobility and mortality (Zuccolo et al., 2023).

Khoury et al. (2021) also reported an increase in EBP in a group of Canadian children aged 7 to 9 years (*n* = 68), during the pandemic with statistically significant differences compared to the pre-pandemic period (internalizing problems: t = 6.46, *p* < 0.001; and externalizing problems: t = 6.13, *p* < 0.001). Ravens-Sieberer et al. (2022) also found a higher occurrence of EBP in a group of children and adolescents, between 7 and 17 years old in Germany (*n* = 1553). Comparing data with a national cohort measured before the COVID-19 pandemic, the prevalence of EBP increased from 9.9% to 17.8% (Ravens-Sieberer et al., 2022). The results of this study also showed that children and adolescents with low socioeconomic status, migration history, or who lived in a household with less than 20 square meters of living space per person were significantly more impacted by the pandemic than their peers, presenting more problems with total mental health (*p* < 0.001), emotional problems (*p* < 0.001), conduct problems (*p* < 0.001), hyperactivity (*p* < 0.001) and peer relationship problems (*p* < 0.001) according to parents’ report on the Strengths and Difficulties Questionnaire (SDQ) (Ravens-Sieberer et al., 2022).

The interruption of in-person classes was associated with an increase in EBP, increased screen exposure, irregular sleep patterns and a decrease in physical exercise (Almeida et al., 2022; Chaabane et al., 2021; Scarpellini et al., 2021). In a study by Scarpellini et al. (2021), 60.2% of caregivers from a sample of Italian children (7 to 13 years old) reported behavioral changes in their children during the remote learning period, such as increased restlessness, aggression and anxiety symptoms, as well as difficulties in paying attention in class for more than 20 min. There is evidence that during the pandemic period, EBP among children and adolescents was associated with lower academic achievement in online classes (Zengin et al., 2021), as well as a decreased interest in developing learning skills, elevated levels of anxiety, excessive worries, and agitation in students (Zhao et al., 2020). Brazil, Argentina, the United States, India, and Mexico were countries with the longest interruptions of in-person classes, with their schools being closed for more than 70 weeks (United Nations Educational, Scientific and Cultural Organization [UNESCO], 2022). In this context, the return to in-person classes was a challenging time for students and teachers (Fundo das Nações Unidas para a Infância [UNICEF], 2020; Wang et al. 2021a).

In a study carried out after the reopening of schools in China, Wang et al. (2021b) observed that 62.51% of children reported fear of falling behind in their class content and 45.12% had prosocial behavior classified in the “borderline” or “clinical” ranges according to parental reports in the SDQ. Another study conducted in China compared EBP in children and adolescents who had started attending face-to-face classes and students who were still studying at home, finding more behavioral problems in the first group (Wang et al. 2021a). Among children aged 6 to 11, it was found that those in-person education had higher scores on the CBCL/6–18 for total problems (*p* = 0.02), internalizing problems (*p* = 0.02) and externalizing problems (*p* = 0.02) than their homeschool peers (Wang et al. 2021a). In Brazil, Assis and Conceição (2023) analyzed the repercussions of the pandemic on the return to in-person classes in students between 8 and 11 years old (*n* = 35) and identified the presence of depressive symptoms in 34.4% of the students on the Child Depression Inventory (CDI) and anxiety symptoms in 57.5% on the Multidimensional Anxiety Scale for Children (MASC). Moreover, qualitative analyses of interviews with parents (*n* = 19) and teachers (*n* = 6) revealed that signs of anxiety were also observed by them in these students (Assis & Conceição, 2023).

Children’s EBP had negative impacts on their social and behavioral adjustment upon returning to school (Akiyoshi et al., 2023; Assis & Conceição, 2023; Meherali et al., 2021; Ye, 2020). This was, and continues to be, a challenging scenario for teachers, as they not only need to be aware of the negative impact of COVID-19 on children’s mental health (Liu et al., 2020), but also need support to manage EBP, enabling the optimization of teaching-learning processes in the classroom context. In this sense, the adoption of school-based intervention programs has generated positive results in improving academic performance indicators and students’ behavioral repertoires (Bolsoni-Silva et al., 2021; Khoury, 2013; Paiano et al., 2019; Raval et al., 2019).

Some of the most used strategies in school-based intervention programs with basic education teacher training include, for example, learning rules, functional analysis, behavior modification, strategies for reducing and eliminating inappropriate behaviors in the classroom context, and promoting social behavior in the school context (Abreu et al., 2015; Cardoso, 2015; Cintra & Del Prette, 2019; Nazar et al., 2020). One of the approaches that has provided the most support and empirical evidence of effectiveness in teacher training to optimize student management strategies is Applied Behavior Analysis (ABA).

ABA-based programs are defined by the application of behavioural analysis techniques, such as functional behavior assessment, reinforcement of desirable behaviors and reduction of behavior problems by positive reinforcement. Furthermore, they emphasize the promotion of skill generalization across varying contexts, supporting behavior change in the long-term. In the school setting, they require teachers to be trained in functional assessment and in function-based behavior support plans, with support and supervision (Grey, 2005). Previous studies have been conducted by implementing teacher training programs based on ABA and other theoretical approaches, using for example, stimulation of communication, initiating and maintaining conversation skills, expression and comprehension of positive and negative feelings, improvement of quality on the teacher-student relationship, and educational social skills (Bolsoni-Silva et al., 2021; Cognetti & Bolsoni-Silva, 2025; Inoue & Takagi, 2021; Nazar et al., 2020). In this study we replicated to Brazilian context the program developed by Inoue and Inoue (2023), a teacher training based on ABA, addressing theoretical aspects of characterizing behavioral problems, functional analysis, differential reinforcement, and stimulus control.

Inoue and Inoue (2023) found a reduction in EBP in a group of 21 children with a diagnosis or suspicion of having autism spectrum disorder (ASD) and attention deficit/hyperactivity disorder (ADHD), after implementing an intervention program with teachers based on ABA principles, such as functional analysis and differential reinforcement to manage student behaviors. A similar result was observed after the implementation of teacher training carried out remotely through online PowerPoint lectures on the use of ABA strategies to manage EBP (Inoue & Takagi, 2021). In the study, six of the seven participating teachers reported a decrease in the EBP in the children assessed after the intervention (Inoue & Takagi, 2021).

Bolsoni-Silva et al. (2021) study revealed a decrease in EBP and an improvement in academic performance in children in a study with elementary school students after the implementation of PROMOVE-Professores/PROMOVE-Teachers, an intervention program aimed at developing educational social skills in teachers that encompassed topics such as expressing positive and negative feelings, skillful and unskillful behaviors, and establishing rules and limits. In the study conducted with elementary school teachers and their students (*n* = 11) with EBP, according to the teachers’ perception, a decrease in EBP was observed in the children as measured by the Teacher’s Report Form (TRF/6–18) and an improvement in students’ academic performance in the post-intervention phase compared to data collected initially.

Another teacher training program was implemented by Nazar et al. (2020) in a sample of 7 teachers and 32 students from elementary school, covering topics such as: principles of behavior analysis and learning, responsiveness, rules, consequences for appropriate and inappropriate behaviors, functional analysis, educational social skills and teacher leadership styles. In the study, based on students’ assessment of teachers’ behavior regarding responsiveness, demandingness, and use of coercive control with repeated measures (pre- and post-intervention), the authors identified an increase in authoritative and authoritarian practices and a reduction in negligent and permissive practices (Nazar et al., 2020). The authors hypothesize that some of the students who identified their teachers as negligent began to perceive them as authoritarian or authoritative, indicating an increase in the presence of teaching behaviors associated with the demanding dimension. Though it was not verified whether the teachers’ behavioral changes had an impact on student behavior and learning. (Nazar et al., 2020).

ABA-based interventions such as the one conducted in our study, typically have a target population such as children, parents or teachers. Teacher-focused interventions have been associated with a reduction in EBP and promotion of social skills in the intervention group of similar studies, and they are typically conducted by training school teachers in ABA techniques (Elias & Amaral, 2016; Bolsoni-Silva et al., 2021). According to Bolsoni-Silva et al. (2021), teacher-focused interventions should be complementary to those that are focused directly on students, and the implementation of each intervention should be considered according to schools’ and students’ needs.

The increase in EBP in the child population in general during and after the COVID-19 pandemic (Wang et al. 2021a) represents a challenge for all teachers since the return to in-person classes in 2022, whether in an attempt to reduce the learning gaps already identified in previous studies (Realyvásquez-Vargas et al., 2020) or to reduce and mitigate EBP that can act as an additional factor of interference in the quality of children’s learning (Borba & Marin, 2017; Mundy et al., 2017; Pagerols et al., 2022). However, although studies aimed at the development and/or implementation of teacher training programs for behavioral management in the classroom have been developed in recent years, there is still a prioritization of research with teachers of students with atypical development (Inoue et al., 2021; Khoury, 2013; Paiano et al., 2019), with small samples (Bolsoni-Silva et al., 2021) and/or without the use of standardized instruments to assess the effects of the intervention on student behavior (Nazar et al., 2020). Study of systematic review and meta-analysis explored the effectiveness of teacher interventions supporting children with externalizing behaviors based on teacher and child outcomes (Aldabbagh et al., 2022). The authors included into the systematic review 31 papers, showing by the meta-analysis a positive effect of teacher intervention on teacher and child outcome (decrease of externalizing behavior problems and enhanced prosocial behavior). However, these 31 studies not included remote training. With the COVID-19 pandemic, professionals from all areas experienced an opportunity to reduce barriers to the implementation of mental telehealth programmes (Lyzwinski et al., 2024). In this context of evidence-based assessment, this study was developed, opting for the use of an online teacher training program. The objective of the present study was to implement and evaluate the effects of an online behavioral teacher training program (based on ABA) to promote pro-social behaviors and improve the EBP of elementary students after the return to in-person classes following the COVID-19 pandemic.

## Method

### Study design

This is a study with a quasi-experimental design with an intervention group (IG) and a control group (CG). The project was approved by the Research Ethics Committee of the Universidade Presbiteriana Mackenzie (CAAE: 53413721.0.0000.0084). The study was authorized by the Municipal Department of Education of the municipality of Embu das Artes, upon signature of the Informed Consent Form (ICF). All school coordinators, teachers, and parents/caregivers signed the ICF prior to data collection. No assent form was obtained from the children, as they did not complete any instruments.

#### Participants

The sample was non-probabilistic, comprising 2nd and 3rd year elementary students and their school teachers from the public education network in the city of Embu das Artes, in the state of São Paulo, Brazil, as well as the parents/caregivers of the students. Teachers were invited to participate in the study through the Municipal Secretary of Education of the city of Embu das Artes. Sixty-four teachers agreed to participate in the study, of which eight did not meet the inclusion criteria as they did not teach in the 2nd or 3rd year, and 19 withdrew from participating before the intervention.

**Fig. 1:**
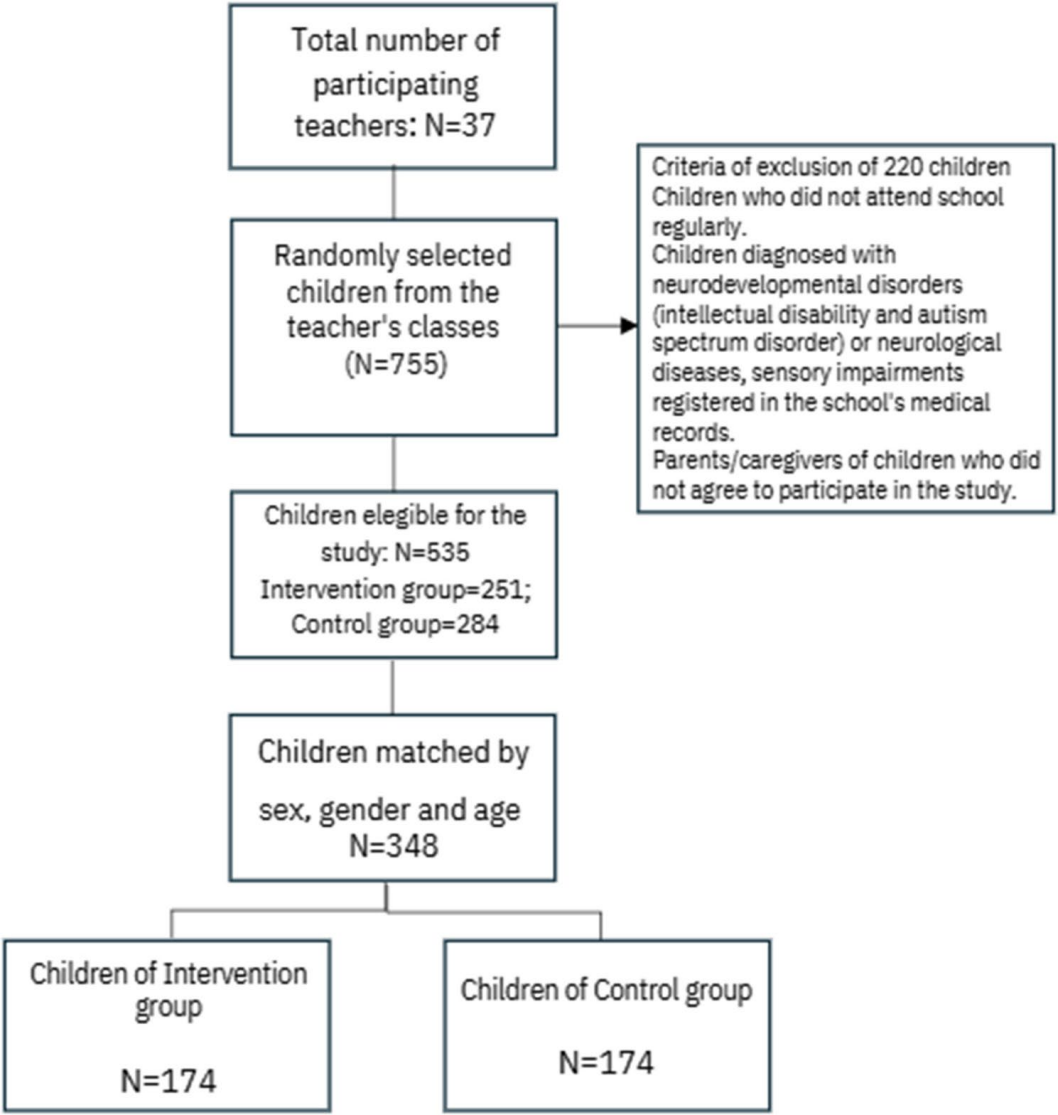
Flowchart of the study sample selection

For this study, the sample was composed by 37 teachers who teach in 15 elementary schools at the municipality. They were randomly distributed into the groups (16 teachers for intervention group and 21 for control group). The distribution of teachers into groups was randomized until there were no teachers from different groups in the same school. The students’ parents/caregivers were selected based on the number of students in each class. All parents/caregivers were selected when there were 21 or fewer students in the class. In classes with more than 21 students, 21 children were selected randomly from the attendance list, resulting in a total of 755 parents/caregivers of selected students. However, of the 755 children who were eligible for the study, 220 children were excluded because they did not meet the inclusion criteria, namely: (a) children who did not attend school regularly, i.e., did not attend the class daily; (b) children diagnosed with neurodevelopmental disorders (intellectual disability and autism spectrum disorder) or neurological diseases, sensory impairments with medical records in the school’s medical records; (c) parents of children who did not agree to participate in the study. Of the 535 eligible parents/caregivers, 251 were parents/caregivers of students from the IG teachers’ classes and 284 from the CG teachers’ classes. Subsequently, 174 students matched by gender, age and school grade were selected from each group, resulting in 348 children (IG = 174 and CG = 174), whose parents/caregivers and teachers responded to the assessment instruments. This procedure was carried out in an R environment using the MatchIt package version 4.5.4. The study included a total sample of 37 teachers, 348 students, and 345 parents/caregivers of these students. Figure 1 presents a flowchart of the sample selection process.

Table 1 presents the sociodemographic characteristics of the parents/caregivers and student participants. In the CG, 84.4% of the respondents are mothers, with an average age of 35.5 years, with the majority having completed high school (53.2%). Similarly, in the IG|, 85.5% are mothers, with an average age of 34.3 years, with the majority’s having completed high school (55.2%). The homogeneity of the CG and IG was tested using the Chi-square test and Student’s T Test in relation to the students’ age, sex and grade, and family socioeconomic level and parents’ education level. No statistically significant differences were found between the groups.

**Table 1:** Sociodemographic characteristics of the sample of students (*N* = 348) and their parents/ caregivers (*N*=345^a^)

Parents/caregivers (*n* = 345)		CG (*n* = 173)	IG (*n* = 172)	*p* value
		*n* (%)	*n* (%)	
Relation to the child	Biological mother	146 (84.4)	147 (85.5)	
	Biological father	18 (10.4)	16 (9.3)	
	Grandmother/grandfather	4 (2.3)	5 (2.9)	
	Aunt Uncle (≥ 18 years)	0 (0.0)	2 (1.2)	
	Sister brother (≥ 18 years)	2 (1.2)	1 (0.6)	
	Stepmother stepfather	3 (1.7)	1 (0.6)	
Age of responding caregiver	Average	35.5	34.3	
	Standard deviation	8.07	7.06	
Marital status of caregiver	Married/with partner	104 (60.1)	107 (62.2)	
	Single	44 (25.4)	54 (31.4)	
	Separated/divorced	20 (11.6)	9 (5.2)	
	Widow	5 (2.9)	2 (1.2)	
Educational level of caregiver	Illiterate	3 (1.7)	1 (0.6)	*p* = 0.50
	Elementary School	40 (23.1)	32 (18.6)	
	High school	92 (53.2)	95 (55.2)	
	Higher education	38 (22.0)	44 (25.6)	
Socioeconomic level	Upper Class (Class A)	3 (1.7)	2 (1.2)	*p* = 0.84
	Upper Middle Class (Class B)	32 (18.5)	31 (18.0)	
	Lower Middle Class (Class C)	110 (63.5)	118 (68.6)	
	Low and Extremely Low Class (Class D and E)	28 (16.2)	21 (12.2)	

Children (*n = 348)*		CG (*n = 174)*	IG (*n* = 174)	
Sex		n (%)	n (%)	1.00
	Female	86 (49.4)	86 (49.4)	
	Male	88 (50.6)	88 (50.6)	
School grade	2nd year	110 (63.2)	110 (63.2)	1.00
	3rd year	64 (36.8)	64 (36.8)	
Age	Mean	7.72	7.72	1.00
	Standard deviation	0.69	0.69	
Use of mental health services	No	163 (93.7)	159 (91.4)	
	Yes	11 (6.3)	15 (8.6)	

Intervention group *IG*, Control group *CG*

^a^The difference between the number of parents and the number students is due to the fact that three parents are responsible for two students each

### Instruments

- Strengths and Difficulties Questionnaire (SDQ-P4-17 – parental version and SDQ-T4-17 – teacher version): the questionnaires assess the emotional and behavioral problems and social skills of children and adolescents aged 4 to 17 years based on the report of parents/caregivers and teachers. The instrument consists of 25 items distributed in five subscales of 5 items each: emotional problems, conduct problems, hyperactivity/inattention, peer relationship problems and pro-social behavior. The score of the first four scales added together generates the total difficulties score (20 items). The questionnaire contains an impact supplement that measures the chronicity of EBP, damage to the child, and the burden on the family. The scale scores are classified into the “normal”, “borderline” and “abnormal” ranges. The instrument presents evidence of content validity with cultural adaptation for the Brazilian population by Fleitlich et al. (2000).
- Parents’ sociodemographic characterization questionnaire: The questionnaire ascertains the educational level of the responding parent/caregivers, their age, relationship with the child (mother/father, grandparents or other type of caregiver) and marital status of the caregiver.
- Brazilian Economic Classification Criteria: A questionnaire developed by the Associação Brasileira de Empresas de Pesquisa [ABEP] that assesses family socioeconomic classification based on the level of education of the head of the family, access to public services and whether the family possesses various household items (classes: A, B1, B2, C1 C2 or DE) (ABEP), 2021).
- Registration checklist: A form used to monitor the implementation of the strategies developed in the IG training program, consisting of 11 items: (1) Establish relationships between behavior, antecedent, and consequence; (2) Praise and highlight positive aspects of student behavior; (3) Use immediate feedback, that is, act quickly after a behavior; (4) Describe and analyze desirable student behaviors; (5) Establish rules with students, ensuring they are accepted; (6) Encourage self-control and self-awareness through activities in which students assume responsibility; (7) Intersperse necessary (less attractive) activities with more attractive ones; (8) Describe undesirable behaviors and point out alternatives; (9) Expand opportunities for interaction in the classroom through group activities; (10) Express attention to appropriate student behavior; 11. Avoid expressing attention to inappropriate student behavior. Teachers were asked to complete the form according to the frequency with which a given strategy was used with students during the indicated period. Scores were as follows: “2” = used often (strategy used daily in class), “1” = used rarely (strategy used only in some classes or on some days), or “0” = not implemented (strategy not used in class). IG teachers completed the registration checklist every 15 days, reporting the application of the strategies in the classroom.

### Intervention group teacher training program

The training program adopted some guidelines from the study by Inoue et al. (2021). The training topics for teachers are described in Table 2. The duration of the training was 12 consecutive weeks between the months of September and November 2022. It was conducted remotely through the digital platform Google Drive, with six video classes lasting between 15 and 20 min, recorded in PowerPoint format. Teachers received one video lesson per week during the first two months and participated in online meetings every fifteen days until the end of the training, totaling five meetings. The meetings took place on the Google Meet platform and aimed to review the content of the video classes, clarify doubts about the content of the video classes and highlight the guidelines to encourage appropriate behavior and manage EBP in the classroom. The contents of the video classes used can be viewed in the supplementary document (Supplementary Material A).

**Table 2:** Themes and contents covered in the training program with the intervention group and the control group

Group	Theme	Content
Intervention	Emotional and behavioral problems in childhood	Definition of emotional and behavioral problems (EBP) (internalizing and externalizing); main factors associated with EBP in childhood; impacts of the COVID-19 pandemic; EBP that can be observed at school when returning to in-person classes.
	Functional behavior analysis	ABC of behavior (Antecedent, Behavior & Consequence): definitions and examples.
	Contextual modification: antecedents and reinforcers	Important antecedents in the school context; reinforcing consequences: natural vs. arbitrary; positive and negative reinforcement; examples of positive reinforcement to use in the classroom.
	Acquisition and maintenance of new behaviors	Modeling behaviors appropriate to the school context; modeling: how the teacher and peers can be models of prosocial behaviors; how to establish and encourage rule following.
	Dealing with inappropriate behavior	Extinction and differential reinforcement to deal with inappropriate behaviors; mediation of conflicts between students; strategies to prevent problems from arising (rules and positive reinforcement).
	Strategies to encourage appropriate behaviors	How to deal with inattention/hyperactivity problems and help students concentrate in class; review with examples of concepts and content covered in previous videos.
Control	Challenges and strategies for reading	The National Law of Guidelines and Bases for Education; the impact of the pandemic on reading skills; reading strategies.
	Pre-reading strategies	Creating goals for reading; prediction and updating of previous knowledge; making predictions; and formulating questions.
	Reading strategies	Shared reading; independent reading; cycle: read, summarize, ask for clarification and predict.
	Post reading strategies	Confirmation or refutation of hypotheses and predictions; discussion of the general idea and creation of materials and reports.

### Control group program

The CG was designed as an active control group and received an alternative intervention focused on strategies and benefits of reading in the classroom (Ferreira & Gonçalves, 2018). Four video lessons were used for the CG, addressing topics such as challenges in stimulating reading in childhood and strategies that can be used before, during, and after reading (e.g., shared reading). The CG themes are presented in more detail in Table 2. The books used and the time dedicated to reading activities with the classes were selected at the discretion of each teacher, so the video lessons presented general strategies for reading in the classroom. The CG lasted 12 weeks, between September and November 2022. The training was delivered remotely through the Google Drive platform, where four video lessons lasting between 13 and 18 min, recorded in PowerPoint format, were made available. In addition, two online meetings were held using the Google Meet platform. The content of the video lessons can be viewed in the supplementary document (Supplementary Material B).

### Data collection procedure

**Fig. 2:**
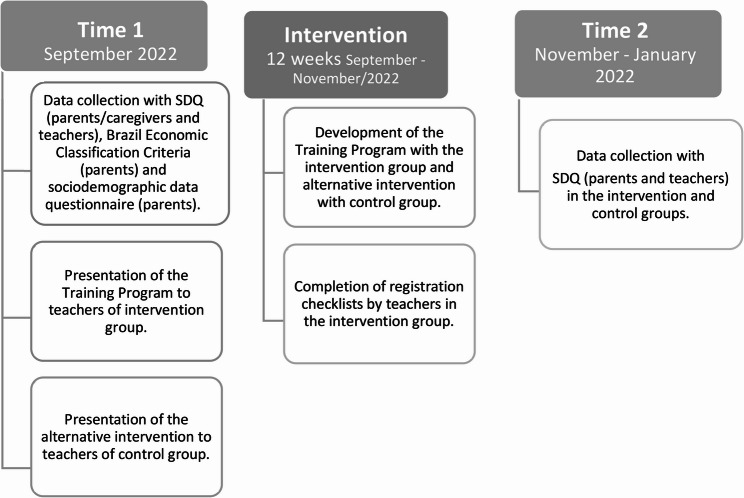
Phases of the study

Before starting to implement the intervention, two online meetings were held separately with each group (IG and CG) to review the study objectives and present the schedule. To implement the intervention, the 16 IG teachers were divided into four groups of four teachers. The 21 GC teachers were divided into three groups of five and one group of six teachers. Meetings with teachers took place during working hours and, if a teacher was unable to participate in the meeting (due to internet connection failures or personal problems, for example) he or she was reassigned to participate in the meeting with another group of teachers from the same group. (IG or CG). Figure 2 illustrates the three phases of the study.

- Pre-intervention (time 1): the SDQ instruments (parents and teachers), the Brazilian Economic Classification Criteria and the sociodemographic characterization questionnaire were applied to both groups (IG and CG). The questionnaires were completed online via the Google Forms platform by all participating teachers and by parents who had a WhatsApp-registered phone number at the school. Printed versions of the questionnaires were sent to parents who did not have a WhatsApp number or who did not receive the messages sent to the provided number. In cases where parents/caregivers had reading difficulties, the questionnaire was administered in person with the assistance of the researcher at the schools. Subsequently, the presentation of the intervention program for the IG and alternative intervention for the GC was carried out. In this phase we received from the teachers 5 completed checklists with a maximum score of 22 points. The low adherence of teachers to completing the checklist in the pre-intervention phase determined that only data from checklists 4 and 5 were included in the study.
- Intervention phase: the duration was 12 weeks, during which teachers from both groups watched the video classes and participated in online meetings in small groups. The IG participants completed the registration checklists.
- Post-intervention phase (time 2): the SDQ instrument (parent and teacher version) was reapplied between weeks 13 and 19.

### Data analysis procedure

Statistical analyses were performed using Jamovi software, version 2.3 and in an R environment using the MatchIt package version 4.5.4. Descriptive analyses were carried out to characterize the sample of parents and students. The Student’s t test was used to compare the SDQ results according to the group condition (intervention or control), at times 1 and 2, according to both informants (parents and teachers). Pearson correlation analysis was performed to verify associations between the SDQ behavioral measures and the use of behavior management strategies by teachers (recording checklists). We initially opted for parametric tests (Student’s t and Pearson’s t) based on the Central Limit Theorem (CLT), assuming that, given our sample size (*N* = 348), the sampling distribution of means would tend toward normality. This provides robustness to these tests even with small deviations from population normality. The value of *p* ≤ 0.05 was adopted for statistical significance, and the values of *p* ≤ 0.06 and *p* ≤ 0.07 were adopted for marginal significance (Dancey & Reidy, 2019). The effect size magnitudes adopted were: ≥0.20 for small effects, ≥ 0.50 for medium effects and ≥ 0.80 for large effects (Cohen, 1992; Dancey & Reidy, 2019). For correlations, values from 0.10 to 0.39 were used for small magnitude, 0.40 to 0.69 for moderate magnitude and above 0.70 for large magnitude (Dancey & Reidy, 2019).

## Results

The main results showed that parents/caregivers and teachers observed a decrease in some indicators of behavioral difficulties in children in the IG. Parents reported significant reductions in conduct problems (*p* = 0.046) and hyperactivity (*p* = 0.039), while teachers noted decreases in hyperactivity (*p* = 0.007) and emotional problems (*p* = 0.043) post-intervention. Greater use of ABA-based strategies by teachers was associated with lower occurrence of emotional and conduct problems in students (*r*=-0.31). No significant reductions were observed in the CG.

### Comparison between times 1 and 2 according to teachers’ reports

According to the teachers’ report at time 1 (pre-intervention phase), the IG had significantly more EBP than the CG on the scales of emotional problems (*p*<0.001), hyperactivity (*p* = 0.024), and peer relationship problems (*p* = 0.006), and in the total score (*p* = 0.001) and impact supplement (p = < 0.001). At time 2 (post-intervention phase), the IG presented significantly more EBP than the CG only in the peer relationship problems (*p* = 0.020) scale and in the impact supplement (*p* = 0.017) (Table 3).

**Table 3:** Intergroup comparison of the control group with the intervention group before and after the intervention

Strengths and Difficulties Questionnaire	Phase	Group	Teachers			Parents		
			Mean	P	D	Mean	p	d
Emotional problems	Time 1	Control	1.586	<.001	-0.378	2.655	0.123	-0.166
		Intervention	2.305			3.034		
	Time 2	Control	1.833	0.324	-0.106	2.782	0.241	-0.126
		Intervention	2.034			3.057		
Conduct problems	Time 1	Control	0.891	0.439	-0.083	1.586	0.047	-0.214
		Intervention	1.029			1960		
	Time 2	Control	0.960	1.000	0.000	1.471	0.197	-0.139
		Intervention	0.960			1.701		
Hyperactivity	Time 1	Control	2.822	0.024	-0.243	3.075	0.002	-0.329
		Intervention	3.477			3.828		
	Time 2	Control	2.701	0.211	-0.134	3.138	0.141	-0.158
		Intervention	3.057			3.506		
Peer relationship problems	Time 1	Control	0.845	0.006	-0.296	1.816	0.420	0.086
		Intervention	1.270			1.678		
	Time 2	Control	0.960	0.020	-0.250	1.707	0.608	0.055
		Intervention	1.345			1.621		
Prosocial behavior	Time 1	Control	7.948	0.829	-0.023	8.397	0.715	0.039
		Intervention	8.006			8.322		
	Time 2	Control	8.489	0.181	0.144	8.374	0.843	-0.021
		Intervention	8.178			8.414		
Total score	Time 1	Control	6.144	0.001	-0.352	9.132	0.032	-0.231
		Intervention	8.080			10.500		
	Time 2	Control	6.454	0.134	-0.161	9.098	0.208	-0.135
		Intervention	7.397			9.885		
Impact Supplement	Time 1	Control	0.195	<.001	-0.422	0.310	0.381	-0.094
		Intervention	0.552			0.385		
	Time 2	Control	0.224	0.017	-0.257	0.241	0.049	-0.212
		Intervention	0.454			0.425		

p= p value

d = Cohen's

According to the report of the IG teachers, the main statistically significant differences were a decrease in EBP between time 1 and 2 reported in the emotional problems (*p* = 0.043) and hyperactivity (*p* = 0.007) scales and in the total score with a marginally significant difference (*p* = 0.065) (Table 4). In the CG, teachers reported significantly higher means at time 2 compared to time 1 on the emotional problems (*p* = 0.059) and prosocial behavior (*p* = 0.002) scales (Table 4).

**Table 4:** Intragroup comparison of SDQ emotional and behavioral problems at time 1 and 2 according to teachers and parents

Strengths and Difficulties Questionnaire scales	Group	Phase	Teachers			Parents		
			Mean	p	D	Mean	P	d
Emotional problems	Control	Time 1	1.586	0.059	-0.144	2.655	0.388	-0.066
		Time 2	1.833			2.782		
	Intervention	Time 1	2.305	0.043	0.155	3.034	0.888	-0.011
		Time 2	2.034			3.057		
Conduct problems	Control	Time 1	0.891	0.467	-0.055	1.586	0.297	0.079
		Time 2	0.960			1.471		
	Intervention	Time 1	1.029	0.525	0.048	1960	0.046	0.153
		Time 2	0.960			1.701		
Hyperactivity	Control	Time 1	2.822	0.528	0.048	3.075	0.666	-0.033
		Time 2	2.701			3.138		
	Intervention	Time 1	3.477	0.007	0.208	3.828	0.039	0.157
		Time 2	3.057			3.506		
Peer relationship problems	Control	Time 1	0.845	0.281	-0.082	1.816	0.394	0.065
		Time 2	0.960			1.707		
	Intervention	Time 1	1.270	0.546	-0.046	1.678	0.649	0.035
		Time 2	1.345			1.621		
Prosocial behavior	Control	Time 1	7.948	0.002	-0.238	8.397	0.884	0.011
		Time 2	8.489			8.374		
	Intervention	Time 1	8.006	0.333	-0.074	8.322	0.583	-0.042
		Time 2	8.178			8.414		
Total score	Control	Time 1	6.144	0.397	-0.064	9.132	0.924	0.007
		Time 2	6.454			9.098		
	Intervention	Time 1	8.080	0.065	0.141	10.500	0.084	0.132
		Time 2	7.397			9.885		
Impact Supplement	Control	Time 1	0.195	0.606	-0.039	0.310	0.175	0.103
		Time 2	0.224			0.241		
	Intervention	Time 1	0.552	0.144	0.111	0.385	0.600	-0.040
		Time 2	0.454			0.425		

p= p value

d =Cohen's

### Comparison between times 1 and 2 according to parents’ reports

According to the parents’ report, the IG had significantly more EBP than the CG at time 1 on the scales of conduct problems (*p* = 0.047), hyperactivity (*p* = 0.002), and total score (*p* = 0.032) at time 1 (Table 3). At time 2, parental data showed no statistically significant differences between groups on the EBP scales. The IG had a significantly higher score than the CG on the impact supplement (*p* = 0.049); however, all significant differences had small effect sizes (Table 3).

The students’ parents reported statistically significant improvements in the IG between times 1 and 2 on the conduct problems (*p* = 0.046) and hyperactivity (*p* = 0.039) scales. According to the CG parents, there were no statistically significant differences between the times (Table 4).

The correlation between the means of the SDQ scales and the use of strategies addressed in teacher training measured by the registration checklists showed that the conduct problems scale (*r*=-0.16) was negatively correlated with the use of strategies according to the registration of the IG teachers in checklist 4. In checklist 5, negative correlations were found between the scales of emotional problems (*r*= -0.32), conduct problems (*r*=-0.19), hyperactivity (*r*=-0.24), relationship with peers (*r*=-0.18) and total score (*r*=-0.31) with the use of behavioral management strategies, indicating that the greater the use of strategies by teachers at the end of the intervention, the lower the occurrence of EBP in the students, mainly emotional problems and total problems (Table 5). Indicators of prosocial behaviors from the SDQ were positively correlated with the use of strategies by teachers in checklist 5 (*r* = 0.25). The correlations between checklist 5 and the hyperactivity, prosocial behavior and total score scales showed small magnitudes (Table 5).

**Table 5:** Correlation between the use of strategies by teachers in the intervention group and the results of the Strengths and Difficulties Questionnaire completed by teachers at time 2

Difficulties Questionnaire scales	Checklist 4	Checklist 5
Emotional problems	-0.13	-0.32**
Conduct problems	-0.16*	-0.19*
Hyperactivity	-0.12	-0.24**
Peer relationship problems	0.11	-0.18*
Prosocial behavior	0.03	0.25**
Total score	-0.11	-0.31**
Impact Supplement	-0.03	-0.04

*<0.05

**<0.001

## Discussion

This study aimed to assess the effects of an online ABA-based teacher training program on the emotional and behavioral patterns of students after returning to classes in person after the COVID-19 pandemic. Studies have revealed an increase in internalizing problems associated with anxiety and depression in children and adolescents during the COVID-19 pandemic and the return to in-person classes (Khoury et al., 2021; Orban et al., 2024; Wang, 2021b). Given this situation, teacher interventions are important to reduce the rates of EBP in children and promote improvements in the mental health landscape (Ford et al., 2019).

The results revealed that after implementing the training program there was an improvement in the students’ behavioral repertoire only in the IG, indicated by a significant reduction in some EBP indicators according to reports of teachers and parents/caregivers of these children. Parents reported significant reductions in conduct problems and hyperactivity, while teachers noted decreases in hyperactivity and emotional problems post-intervention. Studies carried out before the pandemic found an improvement in EBP after developing interventions with educators (Bolsoni-Silva et al., 2021; Cognetti & Bolsoni-Silva, 2025; Fava et al., 2022; Inoue & Inoue, 2023). However, it is noteworthy that there are few studies on teacher training after the COVID-19 pandemic period (An & Zakaria, 2022; Budavari et al., 2025).

Considering the results obtained in the Emotional problems subscale (Table 3), in which no statistically significant differences were found in Time 2 between the IG and CG, we can infer that the main positive effects of the program were on externalizing behavioral problems for this comparison. However, the teachers reported a decrease in emotional problems (*p* = 0.043) post-intervention in the IG (Table 4). The improvement reported by teachers in internalizing problems according to the SDQ in the IG suggests that the training produced positive effects in reducing indicators students’ emotional difficulties assessed by the SDQ, even though the informant was the teacher. Despite this potential limitation about the informant, it is possible to state that the ABA training was effective in the development of strategies to manage the students’ internalizing emotional difficulties, and the results of the current study corroborate the outcomes from previous studies (Allen et al., 2020; Bolsoni-Silva et al., 2021; Cognetti & Bolsoni-Silva, 2025; Corkum et al., 2015; Inoue et al., 2021).

Among the behavioral problems reported in the school context, the occurrence of externalizing problems is usually reported more by teachers compared to internalizing problems (Bolsoni -Silva et al., 2006; Johnson et al., 2023; Silva et al., 2020; Splett et al., 2018). In this study, the externalizing problems reported by teachers who received the training and applied the management strategies on the hyperactivity scale were significantly lower at time 2 compared to time 1. Parents in the IG also reported a decrease in these externalizing problems (hyperactivity and conduct problems) when comparing both times. This indicates that the use of ABA-based behavioral management strategies seems to have led to changes in the behavioral repertoire of students that were generalized to the family context. This result shows the importance of using multiple informants to verify the effects of behavioral intervention programs aimed at children (Achenbach et al., 2017). There are hypotheses that may be associated with the improvement of these emotional problems (predominantly internalizing), as well as hyperactivity (a predominantly externalizing problem). As shown in the study by Inoue and Inoue (2023), it is likely that improvements can be derived from the classroom application of ABA-based program (IG) content, especially functional behavior analysis, the management of contextual factors related to children’s classroom behaviors (antecedents and reinforcers), and strategies to facilitate the acquisition and maintenance of new behaviors.

Although the focus of this study was the development of ABA-based teacher training to teach EBP management strategies (IG), improvements in indicators of students’ prosocial behavior were also reported in the CG after implementing the alternative intervention (a significant increase in prosocial behaviors at time 2). It is possible that the alternative intervention focused on reading provided positive interactions between students and between students and the teacher, promoting the development of these repertoires, especially since one of the phases of the CG’s intervention was focused on reading strategies such as shared reading. Furthermore, reading in the classroom setting promotes discussions, which help to engage students in social interactions. A similar result was reported by Dias-Corrêa et al. (2016), who found a significant increase in prosocial behaviors and a decrease in EBP in a sample of children (*n* = 45) after implementing an intervention program to promote socio-cognitive skills based on reading books. Considering the low reading rates and literacy difficulties in Brazil (Instituto Nacional de Estudos e Pesquisas Educacionais Anísio Teixeira [INEP], 2021), these data are important, as they raise the hypothesis that encouraging reading in the school context, in addition to benefiting academic repertoire, may also stimulate the development of socially competent behaviors, socio-emotional well-being, and social interaction contingencies in students (Weisleder et al., 2018).

There are few studies on the effects of teacher training programs on student behavior using multiple informants, which in this study were teachers and students’ parents (Aldabbagh et al., 2022; Cognetti & Bolsoni-Silva, 2025; Fava et al., 2020). Assessments using multiple informants make it possible to evaluate and understand the generalization of the child’s behavior to different environments (Henz et al., 2021). Furthermore, the use of parents as informants in this study probably reduces methodological bias, since, unlike teachers, parents did not participate in the training. However, there were some discrepancies between the perceptions of these two groups of informants in respect of other SDQ scales. For example, in a scale that assesses internalizing EBP, namely the emotional problems scale, only the teachers reported a statistically significant reduction in mean scores at time 2, while in the conduct problems scale, the reduction was significant only according to the parents’ perception.

Previous studies report different factors that contribute to low rates of agreement between parents and teachers in the assessment of EBP in children (Achenbach, 2020; Achenbach & Ndetei, 2020; Bolsoni-Silva et al., 2018; Bolsoni-Silva & Loureiro, 2021; Carneiro et al., 2021; Cheng et al., 2018), with the most studied factors being those related to parents (Ahmadzadeh et al., 2021; Bai et al., 2022; Gewinger et al., 2022; Pinheiro et al., 2022; Queiroz, 2023; Silva et al., 2019). Including parents in this study as second informants may reduce a possible methodological bias of having only the teacher as an informant in the SDQ, since it was the teacher who received the training and applied the program in the classroom.

It should be noted that the improvement in IG was not the same in all scales, nor in all comparisons between time 1 and time 2. At time 2, parents and teachers in the IG reported higher averages in respect of peer relationship problems, and a greater impact of EBP than in the CG. At time 1, the IG already had higher EBP indicators on the scales of conduct problems, hyperactivity, emotional problems, worse impact indices and a higher average SDQ total EBP score than the CG. However, a positive outcome of this study was that the IG showed an improvement in their EBP, reducing the differences that existed between both groups at time 1 (pre-intervention). Previous studies have found that the use of behavioral management strategies by teachers, such as functional analysis and differential reinforcement can reduce EBP in students (Bolsoni-Silva et al., 2021; Cognetti & Bolsoni-Silva, 2025; Corkum et al., 2015; Inoue et al., 2021).

Functional analysis, defined as the identification of relationships between an individual’s behavior and its consequences, can, in the school context, allow teachers to observe which functional contingencies maintain student behavior (Bolsoni-Silva & Carrara, 2010; Moreira & Medeiros, 2018). This, in turn, allows them to manipulate environmental variables to reinforce behaviors that facilitate learning, such as task completion, and to extinguish undesirable behaviors, such as failure to follow instructions. During the training, teachers were instructed to remain attentive to student behavior and learned to provide reinforcers for appropriate behaviors, while inappropriate behaviors were in the process of extinction. Therefore, it is plausible to hypothesize that, by starting to apply functional analysis to their students’ behavior, they became better equipped to deal with classroom challenges, such as paying more attention to appropriate behaviors instead of undesirable ones (Catania, 1999; Moreira & Medeiros, 2018). In this way, the intervention promoted a DRA (Differential Reinforcement of Alternative Behaviors) procedure: alternative response classes were reinforced, while inappropriate and problematic behaviors did not produce reinforcers. A similar process was observed by Falcão et al. (2016), in a study in which there was also a decrease in behavioral problems after the use of DRA in an intervention to promote social skills and reduce EBP in children.

Another objective of this study was to evaluate the association between the frequency of use of the strategies and the students’ EBP indicators. A statistically significant negative correlation was observed between the use of strategies (checklists 4 and 5) and students’ EBP, according to teachers’ reports at time 2. Similar results were observed in a systematic review and meta-analysis on behavioral interventions carried out with teachers, indicating that the interventions were effective in reducing externalizing behavior problems and symptoms of hyperactivity, as well as improving prosocial behavior (Aldabbagh et al., 2022).

Corroborating these findings, the current study found an association between the use of such management strategies and a lower perception of EBP and a higher level of prosocial behaviors in the children evaluated in the IG. The SDQ prosocial behavior scale encompasses social skills such as empathy, civility and assertiveness. Such skills are protective factors and facilitators of children’s learning and academic success and can be improved in the school context when the teacher provides opportunities for interactions between students and offers appropriate instructions and models of behavior when relating to children (Del Prette and Del Prette 2022a; Prette and Prette 2022b). Changes in teachers’ behavioral management of students (e.g., reduced inappropriate control of student behaviors and increased use of positive reinforcement) appear to have contributed to the development of a greater repertoire of social skills in students. Previous studies have found a positive correlation between the use of negative educational practices, such as aversive control, by teachers and the occurrence of EBP in students, while the presence of social skills in interactions between teachers and students tends to produce a pleasant environment, providing better academic learning and social (Guimarães & Costa, 2023; Mariano & Bolsoni-Silva, 2018; Merle et al., 2022).

Although the results suggest positive effects of ABA-based teacher training on student behavior, these findings should be interpreted with caution due to methodological limitations. The CG and IG students were not homogeneous in terms of emotional and behavioral patterns at time 1, with differences between them in both the reports of the parents and teachers in some of the SDQ EBP scales; the teachers who received the training were the same ones who completed the SDQ about their own students, and we do not control demographic and professional characteristics of the teacher participants; the use of strategies was monitored using checklists that were completed by the IG teachers who had received the training; it was not possible to evaluate training feasibility indicators before carrying out this study, taking into account good implementation practices sciences through a pilot study with the target audience; the low teacher adherence to completing the checklist in the pre-intervention phase determined that only data from checklists 4 and 5 were included in the study. This limitation did not allow a comparison of the behavioral management strategies used by teachers between the pre-intervention and intervention phases; there was no assessment of the informants’ mental health; the control group had access to fewer videos and participated in only two online meetings compared to five in the intervention group; post-test data collection (Time 2) took place between November and early January 2022, a period that coincided with summer vacation in Brazil and may have influenced the perceptions of teachers and parents; the sample size of the number of teachers who participated was relatively small and limited to a single public educational network.

Future studies may involve data collection instruments typical of ABA-based interventions, for example, observation and frequency recording of children’s behaviors in the classroom and comparing these data with data from behavioral assessment instruments based on informant reports. Additionally, the teacher’s management behavior could also be recorded, whether for behavioral problems or for promoting appropriate behavior in children. It is recommended also future replications of this study using observers in the classroom to record student and teacher behavior (in relation to the application of strategies), which will allow a double verification of the fidelity of the implementation of the strategies, as well as the adequacy of the implementation. In this study, there was no record of inappropriate student management strategies in the classroom, for example, the use of aversive control; it is recommended that further studies include this verification to compare with the use of recommended strategies in training. We recommend studies should be undertaken to evaluate the effectiveness of ABA-based teacher training on students in other age groups, such as preschoolers and adolescents, and to consider objective measures and observational data. Furthermore, the present study could be replicated with larger samples and in different settings.

## Conclusion

Measures to contain the COVID-19 pandemic, such as the interruption of in-person classes, created a setting that prompted an increase in children’s EBP. This study aimed to implement and evaluate the effects of an online ABA-based training program for elementary school teachers on the in-person return to classes after the COVID-19 pandemic and the students’ emotional and behavioral management.

Following online intervention with teachers, a significant reduction in some EBP indicators in children was observed, which were perceived by the teachers themselves (hyperactivity and emotional problems scales), and by parents (conduct problems and hyperactivity scales). Although the behavioral strategies were only applied in the classroom, the results showed that the training significantly reduced both the occurrence of internalizing behaviors (emotional problems scale) and externalizing behaviors (hyperactivity scale), with the latter change also being generalized to the family environment. These results indicate that online ABA-based teacher training is a low-cost intervention that produces positive effects on child behavior.

A continuation of this study was initiated in 2023, as a new study with a quasi-experimental design aimed at parental training based on ABA, with the parents of the children who took part in this study. As outcomes, indicators of incremental improvements in the children’s emotional and behavioral patterns are being evaluated. The data from this study is currently being analyzed. The development and implementation of public policies aimed at training teachers in the proper handling of EBP in students in the classroom is a challenge for any school context; however, this challenge has become even more important following the pandemic given the need to reduce the impacts of isolation measures and interruption of face-to-face academic activities on student’s mental health and academic and social development.

## Supplementary Information

Supplementary Material 1

Supplementary Material 2

## Data Availability

The data that support the findings of this study are openly available in Harvard Dataverse at 10.7910/DVN/J4DBEE, reference number V1.

## Abbreviations

COVID: 19–Severe Acute Respiratory Syndrome Coronavirus 2
EBP: Emotional and Behavioral Problems
ABA: Applied Behavior Analysis
ADHD: Attention Deficit/Hyperactivity Disorder
ASD: Autism Spectrum Disorder
UNESCO: United Nations Educational, Scientific and Cultural Organization
UNICEF: Fundo das Nações Unidas para a Infância
INEP: Instituto Nacional de Estudos e Pesquisas Educacionais Anísio Teixeira
CG: Control Group
IG: Intervention Group
SDQ: Strengths and Difficulties Questionnaire
CBCL: Child Behavior Checklist
ABEP: Associação Brasileira de Empresas de Pesquisa
CDI: Children’s Depression Inventory
MASC: Multidimensional Anxiety Scale for Children
