# Supplementary material for: ABA-based teacher training reduces emotional and behavioral problems in Brazilian children
Source: Psicol Reflex Crit. 2026 Mar 25;39:16. doi: 10.1186/s41155-026-00385-2 (PMC13136469; doi:10.1186/s41155-026-00385-2)
Supplement: Supplementary file 1 — Supplementary Material 1 [file 41155_2026_385_MOESM1_ESM.pdf]

Aula 1

Tema de hoje:

Problemas emocionais e comportamentais na infância

Problemas emocionais e comportamentais

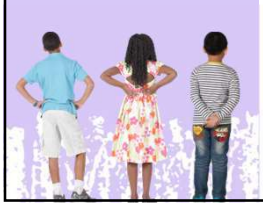

- Problemas emocionais e comportamentais podem ser definidos como excessos ou déficits no comportamento da criança que causam prejuízos para a própria e/ou para as pessoas que convivem com ela.
- Tais problemas podem dificultar o acesso da criança à situações ou oportunidades de se desenvolver e aprender coisas novas.

(Murta, 2007; Leusin, Petrucci & Borsa, 2018)

- Segundo pesquisadores desta área, problemas emocionais e comportamentais na infância ocorrem por um conjunto de fatores de risco da pessoa, da família e do ambiente mais amplo (ex.: escola).

(Murta, 2006)

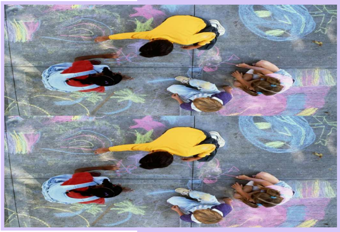

Principais fatores associados a dificuldades emocionais e comportamentais na infância

- Fatores inerentes à criança

Exemplos:
- Características genéticas;
- Problemas de saúde;
- Habilidades/competências sociais da criança.

Principais fatores associados a dificuldades emocionais e comportamentais na infância

- Fatores relativos à família

Exemplos:
- Práticas parentais educativas;
- Demonstrações de afeto;
- Problemas financeiros/conjugais.

Principais fatores associados a dificuldades emocionais e comportamentais na infância

- Práticas culturais

Exemplos:
- Diferenças culturais entre regiões distintas;
- Japão X Brasil;
- São Paulo X Bahia.

Principais fatores associados a dificuldades emocionais e comportamentais na infância

❖ Ambiente escolar

Exemplos:
- Relações estabelecidas entre professores e alunos;
- Relações estabelecidas entre alunos;
- Etc.

Tipos de problemas de comportamento

Tipos de problemas de comportamento

Externalizantes

Afeta predominantemente a(s) pessoa(s) ao redor.

- Ex: Agride outras pessoas.

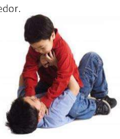

Esta Foto de Autor Desconhecido está licenciado em CC BY-SA-NC

Internalizantes

Afeta predominantemente a própria pessoa.

- Ex.: Sente-se culpada(o) e/ou muito preocupado(a).

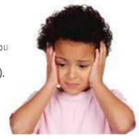

Esta Foto de Autor Desconhecido está licenciado em CC BY-SA-NC

Problemas de comportamento externalizantes

Exemplos:

Foge de casa/escola;
Fala palavrões;
Mente;
Ameaça.

Destrói coisas;

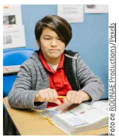

Foto de RODNAE Productions/Pexels

Discute muito, briga, grita;

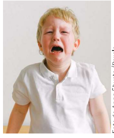

Foto de Anna Shvets/Pexels

Cruel com pessoas/animais;

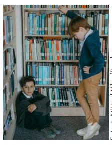

Foto de Mikhail Nilov/Pexels

Não segue regras/desobediente;

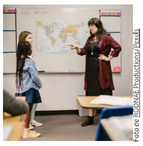

Foto de RODNAE Productions/Pexels

Problemas de comportamento internalizantes

Exemplos:

Sente-se triste;
Cutuca a pele, unhas e outras partes do corpo;
Medos (escola, pessoas...).

Nervoso, medroso, ansioso;

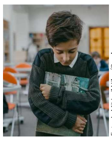

Foto de Mikhail Nilov/Pexels

Poucas coisas lhe dão prazer;

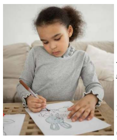

Foto de Monstera/Pexels

Chora muito;

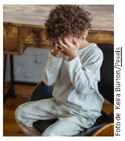

Foto de Keira Burton/Pexels

Reclama que ninguém gosta dele/a;

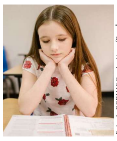

Foto de RODNAE Productions/Pexels

Desatenção/ Dificuldade para se concentrar

Hiperatividade e impulsividade

Exemplos:

Não segue o ritmo da classe;
Não trabalha independentemente;
Não finaliza o que começa.

Olhar parado ('mundo da lua');

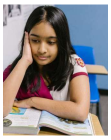

Foto de RODNAE Productions/Pexels

Atrapalha com barulhos excessivos;

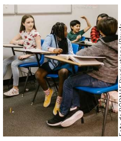

Foto de RODNAE Productions/Pexels

Levanta-se da cadeira frequentemente;

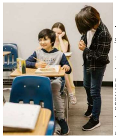

Foto de RODNAE Productions/Pexels

Dificuldade para se concentrar;

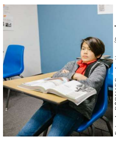

Foto de RODNAE Productions/Pexels

Saúde mental infantil:

Pré-Pandemia e Pandemia da COVID-19

Saúde mental infantil: Pré-Pandemia e Pandemia da COVID-19

PRÉ-PANDEMIA

- Prevalência de transtornos psiquiátricos infanto-juvenis=13,4%
- Ansiedade =6,5%
- Depressão=2,6%

(Polanczyk et al., 2015)

PANDEMIA

- A pandemia COVID-19 afetou o bem-estar social e emocional da infância.

(Brooks et al., 2020; Fiorillo et al., 2020)

- Estudo Canadense (7 a 9 anos)
- Aumento de problemas internalizantes e externalizantes.

(Khoury et al., 2021)

Dificuldades emocionais e comportamentais que podem ser observadas na escola

❖ **Mudanças emocionais**

❑irritabilidade
❑sentimentos de desesperança
❑reações de raiva
❑euforia excessiva
❑tristeza
❑depressão
❑sentimentos de solidão
❑medos excessivos
❑estresse
❑choro
❑rir em demasia

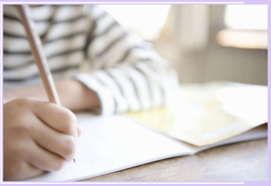

Dificuldades emocionais e comportamentais que podem ser observadas na escola

❖ **Mudanças de comportamento**

❑birras
❑explosões emocionais
❑comportamentos agressivos
❑déficit de assertividade
❑timidez
❑retraimento
❑agitação
❑afastamento de relacionamentos com pares
❑desafios, oposição
❑procura exagerada de socialização
❑sonolência
❑falta de apetite ou comer excessivo
❑preocupação excessiva por contaminação e morte
❑desmotivação pelas atividades escolares
❑comportamentos de risco
❑dificuldades para aderir a regras e rotinas

Como lidar com problemas emocionais e comportamentais na sala de aula?

Observar e acolher

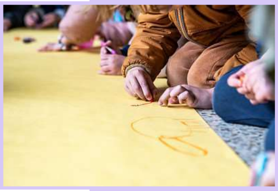

Referências

BROOKS, S. K. et al. The psychological impact of quarantine and how to reduce it: Rapid review of the evidence. *The Lancet*, v. 395, n. 10227, p. 912–920, 26 fev. 2020.

FIORILLO, A.; GORWOOD, P. The consequences of the COVID-19 pandemic on mental health and implications for clinical practice. *European Psychiatry*, v. 63, n. 1, 2020.

Khoury, J. E., Kaur, H., & Gonzalez, A. (2021). Parental Mental Health and Hostility Are Associated With Longitudinal Increases in Child Internalizing and Externalizing Problems During COVID-19. *Frontiers in Psychology*, 12.

LEUSIN, Joanna Ferreira; PETRUCCI, Giovanna Wanderley; BORSA, Juliane Callegaro. Clima Familiar e os problemas emocionais e comportamentais na infância. *Rev. SPAGESP*, Ribeirão Preto, v. 19, n. 1, p. 49-61, 2018.

MURTA, S. G. Programas de prevenção a problemas emocionais e comportamentais em crianças e adolescentes: lições de três décadas de pesquisa. *Psicologia: Reflexão e Crítica*, v. 20, n. 1, p. 01–08, 2007.

POLANCZYK, G. V. et al. Annual Research Review: A meta-analysis of the worldwide prevalence of mental disorders in children and adolescents. *Journal of Child Psychology and Psychiatry*, v. 56, n. 3, p. 345–365, 3 fev. 2015.

Universidade Presbiteriana Mackenzie

Obrigada!

Informações de contato:
rayrassouza07@gmail.com
(11) 9 9237-8267

Rayra Santos de Souza
Maria Cristina Triguero Veloz Teixeira

Aula 2

Tema de hoje:

Análise funcional do comportamento

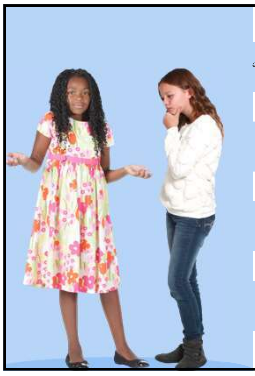

"Nasceu assim"

"Puxou fulano"

"Faz porque quer"

"É isso/é aquilo"

Por que as crianças fazem o que fazem?

"A criança bagunça porque é bagunceira"

"A criança é bagunceira porque bagunça"

O que já sabemos?

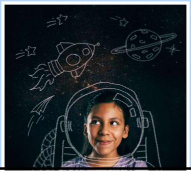

Fatores que influenciam o comportamento das crianças:

- Fatores inerentes à criança (ex.: características genéticas, competências sociais da criança);
- Fatores relativos à família (ex.: práticas parentais educativas, demonstrações de afeto);
- Práticas culturais (ex. diferenças culturais entre regiões distintas);
- Ambiente escolar (ex.: relações estabelecidas entre professores e alunos).

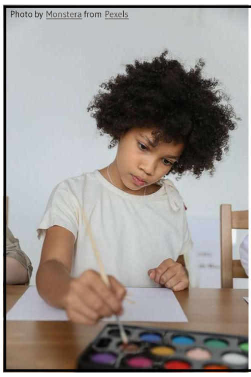

Photo by Monstera from Pexels

Entendendo o comportamento das crianças

Por que as crianças se comportam e como manejar seus comportamentos?

ABC: **A**ntecedent, **B**ehavior & **C**onsequence
(Antecedente, Comportamento e Consequência)

Entendendo o comportamento das crianças

Análise Funcional

A — *Antecedent*
Antecedente: É aquilo que acontece no ambiente logo antes do comportamento.

B — *Behavior*
Comportamento: É tudo aquilo que uma pessoa faz ou diz.

C — *Consequence*
Consequência: É o que ocorre no ambiente logo depois do comportamento.

A

Antecedent

Antecedente

Como identificar os antecedentes?

- Em qual contexto o comportamento ocorreu?
- Onde aconteceu?
- Quem estava presente?
- Quando aconteceu?
- O que foi falado imediatamente antes do comportamento acontecer?

A	B	C
- Horário do recreio; - Som do sinal da escola.	Maria pergunta se já pode sair para o recreio.	Professora autoriza as crianças a saírem as sala.

B

Behavior

Comportamento

Como identificar o comportamento?

- É tudo aquilo que uma pessoa faz, seja observável ou não;
- É como um verbo, é ativo;
- É uma ação e não uma característica da pessoa.

A	B	C
Professora explica como fazer uma tarefa.	Camilo faz a tarefa.	Professora parabeniza Camilo.

C

Consequence

Consequência

Como identificar as consequências?

- O que aconteceu imediatamente após o comportamento?
- O que foi falado?
- A criança conseguiu algo que queria?
- A criança foi punida?

A	B	C
Professora conta uma história para a turma.	Antônio fala alto sobre um assunto diferente.	Professora para a história e conversa com Antônio.

Por que a consequência é importante?

- O comportamento produz consequências no ambiente;
- O comportamento é afetado (controlado) por suas consequências.

A	B	C
Professora conta uma história para a turma.	Antônio fala alto sobre um assunto diferente.	Professora para a história e conversa com Antônio.

Por que a consequência é importante?

- Boas consequências: tendem a aumentar a probabilidade daquele comportamento acontecer novamente em uma situação parecida.
- Consequências aversivas: podem diminuir a probabilidade daquele comportamento acontecer novamente, mas produzem efeitos colaterais indesejáveis. Ex.: choro, grito, mentira.

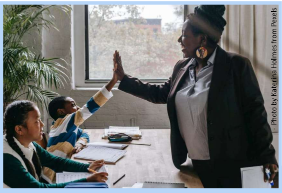

Treino de análise funcional: ABC

A	B	C
Antecedente	Comportamento	Consequência

Treino de análise funcional: ABC

A	B	C
Antecedente	Comportamento	Consequência
Professora pede para que Ana leia sua resposta para o restante da turma.	Lucas lê sua resposta rapidamente em voz alta no lugar da colega.	Professora repreende Lucas dizendo para ele ficar quieto e deixar de ser mal educado.

Treino de análise funcional: ABC

A	B	C
Antecedente	Comportamento	Consequência
Professora pede para que Ana leia sua resposta para o restante da turma.	Lucas lê sua resposta rapidamente em voz alta no lugar da colega.	Professora diz que Lucas acertou e Ana pode responder a próxima pergunta.

Treino de análise funcional: ABC

A	B	C
Antecedente	Comportamento	Consequência
Professora pede para que Ana leia sua resposta para o restante da turma.	Lucas lê sua resposta rapidamente em voz alta no lugar da colega.	Professora explica para Lucas que é preciso respeitar a vez dos colegas, pede para que Ana ainda leia sua resposta e, posteriormente, oportuniza que Lucas também leia uma resposta para a turma.

Se o comportamento das pessoas é influenciado por suas consequências podemos modificar seus comportamentos planejando consequências especiais para eles.

(Moreira & Medeiros, 2007)

Referências

KHOURY, L. P. et al. Manejo comportamental de crianças com Transtornos do Espectro do Autismo em condição de inclusão escolar: guia de orientação a professores. São Paulo: Memnon, 2014.

MOREIRA, M. B.; MEDEIROS, C. A. *Princípios básicos de análise do comportamento*. Porto Alegre (RS): Artmed, 2007.

Universidade Presbiteriana Mackenzie

Obrigada!

Informações de contato:
rayrassouza07@gmail.com
(11) 9 9237-8267

Rayra Santos de Souza
Maria Cristina Triguero Veloz Teixeira

Aula 3

Tema de hoje:

Modificação contextual: Antecedentes e reforçadores

Recapitulando...

A — *Antecedente:* É aquilo que acontece no ambiente logo antes do comportamento.

B — *Comportamento (Behavior):* É tudo aquilo que uma pessoa faz ou diz.

C — *Consequência:* É o que ocorre no ambiente logo depois do comportamento.

✓ O comportamento não ocorre no vácuo, sempre ocorre em um contexto (lugar, horário, a presença de alguém, etc.).

✓ O comportamento das pessoas é influenciado por suas consequências, por isso podemos modificar seus comportamentos planejando consequências.

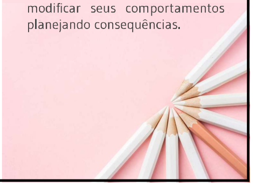

Antecedentes importantes na sala de aula

Discriminação
Em qual ocasião o comportamento ocorreu?

Alguns sinais do ambiente demonstram que um comportamento provavelmente trará uma consequência boa um ruim.

Exemplo:

A		B		C
A professora ensina a fazer "meditação com gargalhada".	➡	*As crianças gargalham fazendo barulho.*	➡	*A professora ri e diz "Muito bem!".*

A atividade "meditação com gargalhada" sinaliza que gargalhar e fazer barulho trará uma consequência boa (sorriso e aprovação da professora).

Antecedentes importantes na sala de aula

Privação e saciação
Quando a consequência vale a pena?

Existem situações nas quais uma consequência boa vai ser mais ou menos valorizada dependendo do acesso que criança já teve àquela consequência.

Exemplo:

A		B		C
- *Não houve aula de Educação Física;*<br>- *A professora combina de fazer uma brincadeira na quadra após a tarefa.*	➡	*As crianças se concentram e fazem a tarefa rápidamente.*	➡	*A professora vai com a turma para o recreio na quadra para brincar de pega-pega, polícia e ladrão etc.*

Não ter tido aula de Educação Física naquele dia aumenta o valor positivo da consequência de ter uma brincadeira na quadra após a atividade.

Foto de Katerina Holmes/pexels

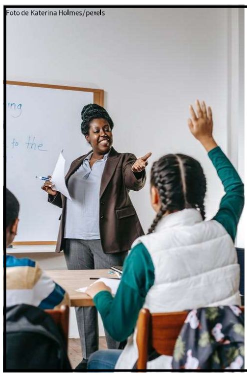

Consequências

- O nosso comportamento é influenciado por suas consequências.

Reforço são consequências que **aumentam as chances** de voltarmos a nos comportar daquela forma.

Consequências reforçadoras podem ser:

Naturais

Quando a consequência reforçadora é um resultado do próprio comportamento.

Ex.: A criança ler sozinha porque é reforçada pela própria leitura.

Foto de Talita Rocha

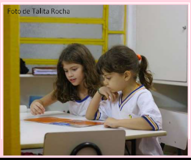

Arbitrárias

Quando a consequência reforçadora é um resultado indireto do comportamento.

Ex.: Ler na escola e ser reforçado pela aprovação da professora.

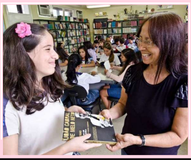

Tipos de consequências reforçadoras

Reforço Positivo

O **acréscimo** de algo no ambiente aumenta as chances do comportamento se repetir.

- Ex.: *Artur pede a ajuda da professora para fazer uma atividade; a professora explica como fazer a atividade; Artur agradece sorrindo: "Brigada, prô!".*

Reforço Negativo

A **eliminação ou a evitação** de algo aumenta as chances do comportamento se repetir.

- Ex.: *Os alunos estão conversando alto e sem prestar atenção na professora; a professora faz o som "pam pararara"; os alunos param de conversar e respondem "pam pam".*

Consequências reforçadoras na sala de aula

- Atenção do professor/a
- Aprovação dos colegas
- Elogios
- Carimbos/adesivos
- Brincadeiras
- Etc.

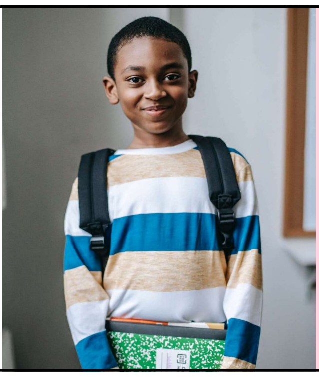

Outro efeito das consequências reforçadoras

Diminuir a frequência de outros comportamentos.

Ex.: *Marcelo frequentemente se levanta e anda pela sala distraindo os colegas; sua professora passa a pedir que ele ajude na aula (entregar atividades, ler exemplos) e elogiá-lo por sua gentileza; A frequência com a qual Marcelo se levanta, anda pela sala e distrai os colegas diminui.*

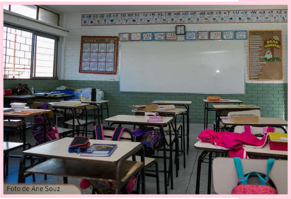

Foto de Ane Souz

Os comportamentos adequados das crianças precisam de consequências reforçadoras sempre ?

Depende!

Consequências reforçadoras sempre: ENSINAR

Consequências reforçadoras às vezes: MANTER

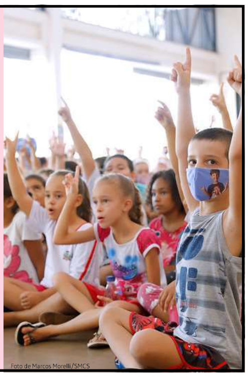

Foto de Marcos Morelli/SMCS

Exemplos: Consequências reforçadoras sempre

- Para ensinar Patrícia a falar em momentos apropriados, a professora parabeniza a menina sempre que ela responde suas perguntas.

- Para ensinar um exercício novo, a professora incentiva dizendo "muito bem" em cada pequeno passo correto que Gabrieli dá.

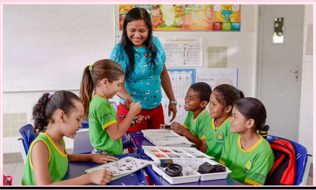

Exemplos: Consequências reforçadoras às vezes

- Lucas frequentemente interrompe a professora para pedir brinquedos em momentos inadequados, geralmente a professora explica que não é o momento e continua com a aula, mas, às vezes, deixa Lucas pegar o que quer para que ele fique quieto.

- Marina já sabe ler e frequentemente se oferece para ler quando a professora pede a ajuda de alguém, por isso a professora às vezes deixa que Marina leia e a parabeniza por seu desempenho e, outras vezes, sugere que Marina deixe outra criança também ler um pouquinho.

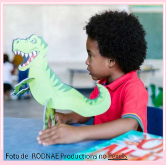

Foto de RODNAE Productions no Pexels

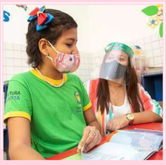

Para não esquecer...

1. **Reforce seus alunos:** usar consequências reforçadoras na sala de aula incentiva as crianças a terem comportamentos adequados;
2. **Observe:** o que é reforçador para algumas crianças, pode não ser para todas as outras;
3. **Reaja rápido:** a consequência reforçadora deve acontecer imediatamente após o comportamento adequado.

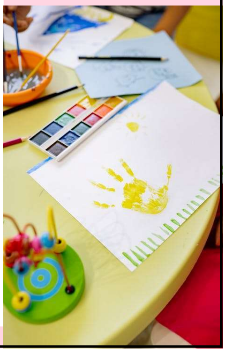

Referências

MENEZES, C. C.; GON, M. C. C. A influência de eventos antecedentes nos problemas de comportamento infantil: revisão de conceitos e aplicabilidade. *Interação em Psicologia*, v. 15, n. 2, 5 jun. 2012. Acesso em: 20 out. 2021.

MOREIRA, M. B.; MEDEIROS, C. A. *Princípios básicos de análise do comportamento*. Porto Alegre (RS): Artmed, 2007.

Universidade Presbiteriana Mackenzie

Obrigada!

Informações de contato:
rayrassouza07@gmail.com
(11) 9 9237-8267

Rayra Santos de Souza
Maria Cristina Triguero Veloz Teixeira

Aula 4

Tema de hoje:

Aquisição e manutenção de novos comportamentos

Aquisição e manutenção de novos comportamentos

Retomando...

- ✓ O comportamento das pessoas é influenciado pelo contexto (antecedentes) e pelas consequências (ex. reforçadores);
- ✓ Reforçadores são consequências que aumentam as chances do comportamento voltar a ocorrer;
- ✓ Fornecer reforçadores sempre após um comportamento adequado é o ideal quando a criança está aprendendo um comportamento novo;
- ✓ Fornecer reforçadores somente às vezes após um comportamento adequado é recomendado para fazer a manutenção de um comportamento que a criança já faz com frequência.

Aquisição e manutenção de novos comportamentos

Nem todo aprendizado precisa acontecer por exposição direta às situações reais. Um novo comportamento pode ser aprendido de três formas:

Aquisição e manutenção de novos comportamentos

Nem todo aprendizado precisa acontecer por exposição direta às situações reais. Um novo comportamento pode ser aprendido de três formas:

1 *Modelagem*
O comportamento é aprendido a partir da exposição direta a uma situação.

2 *Modelação*
O comportamento é aprendido a partir da observação de um modelo.

3 *Regras*
O comportamento é aprendido a partir de uma instrução.

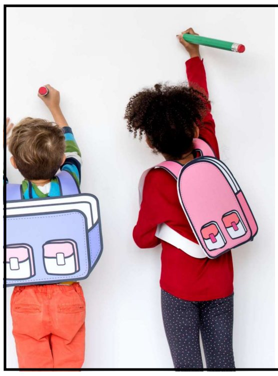

Modelação

Ocorre quando um comportamento é aprendido por meio da observação de um modelo.

- ✓ Ex.: pais, professores, colegas...

Aprendizagem por modelo na escola

Os professores podem oferecer modelos adequados de relacionamento na sua interação com as crianças, que frequentemente observam e copiam os adultos.

E o que o professor pode fazer?

- Seguir seus objetivos quando passa por uma experiência negativa (ex.: manejar e orientar o comportamento de um aluno que lhe gera sentimentos de raiva);
- Utilizar suas emoções/situações de estresse para orientar os alunos, auxiliando-os a nomear e identificar emoções;
- Evitar utilizar punições/castigos/broncas para corrigir problemas de comportamento dos alunos;
- Escutar, demonstrar afeto e incentivo para os alunos.

Aprendizagem por modelo na escola

Del Prette e Del Prette (2013) apresentam exemplos de como os professores podem fazer criticas e elogios que servirão de modelos adequados para os alunos:

Evite dizer assim	Procure falar assim
Você está sempre fazendo coisas erradas para seus colegas.	Quando você se comporta dessa maneira, prejudica seus colegas.
Parabéns, Tiago, você foi o melhor de todos!	Parabéns, Tiago, você se superou nessa tarefa!
Tente ser como o Aguinaldo, um aluno estudioso e bem educado.	Trabalhe um pouco com o Aguinaldo. Cada um pode ajudar o outro com alguma coisa.

(Retirado de Del Prette e Del Prette, 2013, p. 67)

Aprendizagem por modelo na escola

A qualidade das relações dos alunos pode ser mediada pelo(a) professor(a) quando ele(a) oferece modelos adequados, estabelece limites e amplia oportunidades de interação entre as crianças.

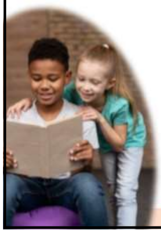

Como incentivar que os alunos aprendam uns com os outros?

- ✓ Oportunizando atividades em duplas estratégicas;
- ✓ Selecionando monitores para a aula;
- ✓ Valorizando as contribuições de todos;
- ✓ Solicitando a ajuda de um(a) aluno(a) para inserir/acolher os novos alunos no grupo.

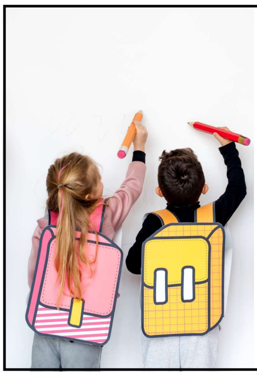

Regra

É um estímulo que antecede o comportamento descrevendo um comportamento esperado e sua provável consequência. A regra oportuniza que as crianças se comportem a partir da instrução do professor.

- ✓ Alguns comportamentos podem ser aprendidos por esse tipo de descrição (ex.: atravessar a rua).
- ✓ Combinados de sala de aula.

Aprendizagem por regras na sala de aula

Como estabelecer regras que as crianças irão seguir?

Ao estabelecer regras, certifique-se de que elas sejam aceitas coletivamente, pois elas devem ser utilizadas como estabelecedoras de limites e relembradas constantemente.

- ✓ Clareza - as regras devem ser diretas e claras e, preferencialmente, combinadas entre os alunos e professor;
- ✓ Coerência - de acordo com a idade;
- ✓ Sistematicidade de rotinas – manter o combinado até o fim;
- ✓ Monitoramento - regras devem ser cumpridas, mas sempre manter exceções.

Dicas para incentivar o seguimento das regras:

- ✓ Sempre que possível lembrar as regras, principalmente, quando o professor percebe que elas podem ser incumpridas;
- ✓ Ficar atento ao clima da sala de aula: muitas vezes é possível observar os sinais e prever quando uma aluno vai se comportar de forma inadequada;
- ✓ "Prevenir é melhor que remediar": utilize as regras e combinados para incentivar que os alunos se comportem de forma adequada.

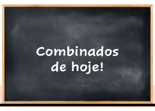

O que fazer quando a criança segue as regras e instruções?

- ✓ Agir rapidamente após um comportamento adequado, propiciando imediato retorno (sem exagerar na expressão verbal ou facial).
- ✓ Motivar a criança evidenciando aspectos positivos do seu comportamento.
- ✓ Utilizar elogios e demonstração de aprovação quando as regras/instruções forem seguidas adequadamente.

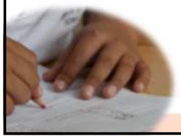

Referências

ARAÚJO, MA. V. *Manejo comportamental pelo professor no contexto de sala de aula de alunos identificados com TDAH.* 2021. Tese (Doutorado em Distúrbios do Desenvolvimento) – Programa de Pós-Graduação em Distúrbios do Desenvolvimento, Universidade Presbiteriana Mackenzie, São Paulo., 2021. Disponível em: <http://tede.mackenzie.br/jspui/handle/tede/1761>. Acesso em: 11 out. 2021.

DEL PRETTE, A., & DEL PRETTE, Z. A. P. (2013). *Psicologia das habilidades sociais na infância: Teoria e prática* (6th ed., p. 280). Editora Vozes.

JUSTO, A. R.; ANDRETTA, I. Competências socioemocionais de professores: avaliação de habilidades sociais educativas e regulação emocional. *Revista Psicologia da Educação*, v. 1, n. 50, 2020. Acesso em: 25 ago. 2021.

NAZAR, T. C. G.; WEBER, L. N. D. Programa de qualidade na interação professor-aluno (PQIPA): descrever para intervir. *International Journal of Developmental and Educational Psychology. Revista INFAD de Psicología.*, v. 2, n. 1, p. 355, 13 jul. 2019. Acesso em: 12 out. 2021.

ROSIN-PINOLA, A. R. et al. Ensinando habilidades sociais educativas para professores no contexto da inclusão escolar. *Revista Educação Especial*, v. 30, n. 59, p. 737, 7 nov. 2017. Acesso em: 19 set. 2022.

Universidade Presbiteriana Mackenzie

Obrigada!

Informações de contato:
rayrassouza07@gmail.com
(11) 9 9237-8267

Rayra Santos de Souza
Maria Clara Neder
Maria Cristina Triguero Veloz Teixeira

Aula 5

Tema de hoje:

Lidando com comportamentos inadequados

Retomando...

- Problemas emocionais e comportamentais atrapalham o processo de ensino-aprendizagem das crianças (PANDEY et al., 2018).
- Intervenções/treinamentos com professores ajudam a diminuir problemas emocionais e comportamentais e melhoram o aproveitamento escolar dos alunos (PANDEY et al., 2018; NAZAR & WEBER, 2019).
- Estratégias para evitar a ocorrência de comportamentos inadequados:
  - Estabelecer regras e combinados e sempre lembrar desses combinados;
  - Elogiar e demostrar sua alegria se os combinados são seguidos, mesmo que você não peça;
  - Ser um modelo para os alunos;
  - Reforçar e incentivar bons comportamentos.

Mas o que fazer quando o problema já aconteceu ?

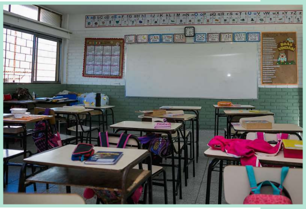

Estratégias para lidar com comportamentos inadequados

1 *Extinção*
Parar de oferecer reforço (atenção, elogios, brincadeiras) para o comportamento *"problema"* até ele ser **extinto**.

2 *Reforçamento diferencial*
Oferecer reforço (atenção, elogios, brincadeiras) para comportamentos **diferentes** do comportamento *"problema"*.

Extinção

O(A) professora(a) **para de oferecer reforço** para o comportamento *"problema"* até ele ser **extinto**.

Exemplos:

	A	B	C
Reforço	- Histórico de dificuldade com as atividades;<br>- Professora passou atividade.	Pedrinho diz que não vai fazer e joga o caderno chão.	- Professora manda Pedrinho para a coordenação;<br>- Pedrinho se livra da atividade.
**_Extinção_**	- Histórico de dificuldade com as atividades;<br>- Professora passou atividade.	Pedrinho diz que não vai fazer e joga o caderno chão.	- Professora conversa com Pedrinho explicando que não é correto jogar o caderno no chão, que a atividade é só um treino e que se ele tiver dúvidas pode perguntar e ela irá ajudá-lo.

Extinção

O(A) professora(a) **para de oferecer reforço** para o comportamento *"problema"* até ele ser **extinto**.

Considerações importantes:

- A extinção de um comportamento inadequado é um **processo**;
- O comportamento pode aumentar de frequência antes de ser extinto;
- Esse processo pode gerar sentimentos de frustração na criança, por isso o(a) professor(a) deve ensinar alternativas de comportamento para conseguir consequências boas.

→ Reforçamento diferencial

Reforçamento Diferencial

O(A) professora(a) **oferece reforço** para comportamentos **diferentes** do comportamento *"problema"*.

Exemplos:

	A	B	C
**_Reforçamento Diferencial_**	- Histórico de dificuldade com as atividades;<br>- Início do processo de extinção;<br>- Professora passou atividade.	Pedrinho pede ajuda para fazer a atividade.	- Professora ajuda Pedrinho e diz que ficou muito feliz/orgulhosa por ele ter pedido ajuda.

Reforçamento Diferencial

Estratégias de reforçamento diferencial:

- Reforçar comportamentos que impedem a ocorrência do comportamento *"problema"*.
  Ex.: Levantar sem motivo X Ficar sentando(a) fazendo uma tarefa.
- Reforçar comportamentos alternativos para o comportamento *"problema"*.
  Ex.: Falar junto com a professora X Levantar a mão antes de falar.
- Reforçar outros comportamentos do aluno após ele passar um tempo predeterminado sem emitir o comportamentos *"problema"*.

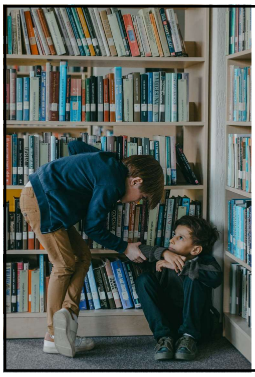

Mediando conflitos

Estratégias para resolver e evitar conflitos entre os alunos.

Estratégias para resolver e evitar conflitos

- Se um aluno(a) iniciar um comportamento inapropriado (ex.: provocar um colega) interrompa imediatamente (ex. solicite que o aluno faça algo);
- Interrompa conflito entre alunos, afastando-os um do outro e envolvendo-os em outras atividades;
- Peça que os alunos envolvidos no conflito contem como se sentem e pensem nas consequências daquele comportamento;
- Oportunize momentos de interação entre os alunos: muitas vezes as crianças podem se envolver em conflitos com os colegas por não saberem interagir de outra forma.
- Observe sempre o ambiente para que possa agir antes do problema de comportamento ocorrer.

Lidando com problemas emocionais e comportamentais

O que fazer?

- Converse com o(a) aluno(a) mostrando outras formas de pensar;
- Peça para o(a) aluno(a) explicitar quais consequências seu comportamento pode ter para ele(a) mesmo(a) e os colegas;
- Dê alternativas de comportamentos mais adequados para a situação;
  (Ex.: pegar borracha no estojo do colega X pedir a borracha emprestada)

Concluindo...

- Antecipe-se aos problemas: Use muitas instruções e se necessário repita os combinados e instruções para que os comportamentos problema não ocorram (ex.: desafios, negativas, pouca colaboração, desmotivação).
- Lembre-se: Comportamentos adequados para a escola também precisam ser ensinados.
- Tenha paciência: Mudanças de comportamento levam tempo.
- Valorize as pequenas conquistas: Demonstre alegria quando aquela criança que costuma apresentar problema de comportamento, se comporta bem ou como você espera!

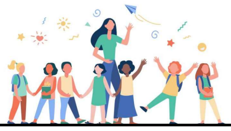

Referências

COGNETTI, N. P.; BOLSONI-SILVA, A. T. *Habilidades Sociais para Professores.* São Carlos, SP: Suprema Gráfica e Editora Eireli. 2019.

Moreira, M. B., & Medeiros, C. A. (2007). *Princípios básicos de análise do comportamento.* Artmed.

NAZAR, T. C. G.; WEBER, L. N. D. Programa de qualidade na interação professor-aluno (PQIPA): descrever para intervir. International Journal of Developmental and Educational Psychology. *Revista INFAD de Psicología.*, v. 2, n. 1, p. 355, 13 jul. 2019.

PANDEY, A. et al. Effectiveness of Universal Self-regulation-Based Interventions in Children and Adolescents: A Systematic Review and Meta-analysis. *JAMA pediatrics*, v. 172, n. 6, p. 566–575, 2018. Disponível em: <https://www.ncbi.nlm.nih.gov/pubmed/29710097>.

ROSIN-PINOLA, A. R. et al. Ensinando habilidades sociais educativas para professores no contexto da inclusão escolar. *Revista Educação Especial*, v. 30, n. 59, p. 737, 7 nov. 2017. Acesso em: 19 set. 2022.

Universidade Presbiteriana Mackenzie

Obrigada!

Informações de contato:
rayrassouza07@gmail.com
(11) 9 9237-8267

Rayra Santos de Souza
Maria Clara Neder
Maria Cristina Triguero Veloz Teixeira

Aula 6

Tema de hoje:

Estratégias para incentivar comportamentos adequados

Retomando...

Tipos de problemas de comportamento

Externalizantes

Afeta predominantemente a(s) pessoa(s) ao redor.

- Ex: Agride outras pessoas.

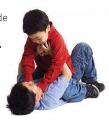

Esta Foto de Autor Desconhecido está licenciado em CC BY-SA-NC

Desatenção, hiperatividade e impulsividade

Internalizantes

Afeta predominantemente a própria pessoa.

- Ex.: Sente-se culpada(o) e/ou muito preocupado(a)

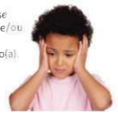

Esta Foto de Autor Desconhecido está licenciado em CC BY-SA-NC

Problemas de desatenção, hiperatividade e impulsividade

Por que os alunos não se concentram na aula?

- Medo de errar (fugir da punição);
- Pouco interesse nas atividades (falta de reforçadores);

Como ajudar os alunos a se concentrarem na aula?

- ✓ Assegurar a criança de que ela vai conseguir fazer as tarefas;
- ✓ Mesclar atividades menos atrativas com as mais divertidas;
- ✓ Comunicar o tempo ao longo da aula;
- ✓ Colocar a criança sentada perto do professor;
- ✓ Se aproximar mais ao se comunicar com a criança;
- ✓ Fracionar a carga de trabalho total.

Aumentando bons comportamentos e diminuindo comportamentos inadequados

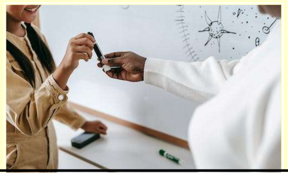

Estratégias para a sala de aula

- ✓ Estabelecer relações entre o comportamento, antecedente e consequência.
  - O que aconteceu antes?
  - O que o aluno fez?
  - O que aconteceu depois?

  A – B – C

- ✓ Descrever e analisar comportamentos desejáveis dos alunos.
  - Explique claramente o que você espera dos alunos;
  - Relembre os combinados sempre que for possível.

  Ex.: Antes de se ausentar da sala, a professora lembra as crianças que os combinados continuam valendo (devem respeitar os colegas e terminar a lição para terem tempo de brincar com a professora quando ela voltar).

Estratégias para a sala de aula

- ✓ Elogiar e evidenciar aspectos positivos do comportamento dos alunos.
- ✓ Usar retorno imediato, isto é, agir rapidamente após um comportamento.
  - Atenção e aprovação da professora;
  - Valorização dos colegas (ex. palmas);
  - Ferramentas lúdicas (ex.: dinheirinho, carinha, carimbo, adesivo);
  - Brincadeiras etc.

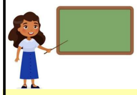

Estratégias para a sala de aula

- ✓ Estabelecer regras com os alunos, certificando-se de que elas sejam aceitas.
  - Sempre relembrar regras e combinados para evitar que o problema aconteça.
- ✓ Estimular o autocontrole e a autopercepção utilizando de atividades nas quais os alunos assumam responsabilidades.
  - Atividades diferenciadas (ex: desenhos, livros etc.);
  - Monitores/ajudantes de sala;
  - Duplas estratégicas.

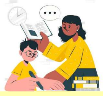

Estratégias para a sala de aula

- ✓ Intercalar atividades necessárias (de baixa atratividade) com atividades mais atrativas (divertidas).
- ✓ Fracionar a carga de trabalho total por meio tarefas mais curtas.

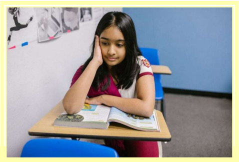

Estratégias para a sala de aula

- ✓ Ampliar as oportunidades de interação na sala de aula, por meio de atividades em grupo.

Ex.: Duplas estratégicas

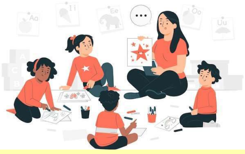

Estratégias para a sala de aula

- ✓ Descrever comportamentos indesejáveis apontando alternativas.
  - Ex.: Se acontecer algo com vocês enquanto eu não estiver na sala (provocação de um colega), não é para vocês levarem isso para casa sem me contar. Vocês devem me contar quando eu voltar para a sala. Ok?
- ✓ Não expressar atenção para comportamentos inadequados dos alunos.
  - Ex: Aluna interrompe a professora para contar histórias que não tem relação com a aula. Professora relembra o tema ou a pergunta da aula e continua a atividade com os alunos, sem expressar atenção exagerada com o aluno que interrompeu.
  - Em outro momento deve-se oportunizar que o aluno conte sua história.
- ✓ Expressar atenção para comportamentos adequados.

Para finalizar...

- ❑ Não esqueça:

Você é uma referência para seus alunos!

O afeto, a atenção, a aprovação, o apoio e o carinho que você transmite ajudará a criança a se comportar de forma adequada na sala de aula e acompanhará ela pelos próximos anos na jornada da aprendizagem.
