# Supplementary material for: ABA-based teacher training reduces emotional and behavioral problems in Brazilian children
Source: Psicol Reflex Crit. 2026 Mar 25;39:16. doi: 10.1186/s41155-026-00385-2 (PMC13136469; doi:10.1186/s41155-026-00385-2)
Supplement: Supplementary file 2 — Supplementary Material 2 [file 41155_2026_385_MOESM2_ESM.pdf]

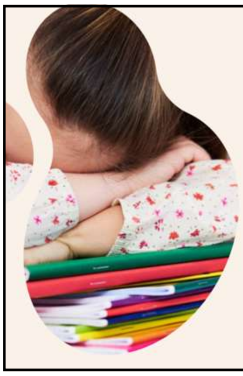

Aula 1

Tema de hoje:

Desafios e estratégias para a leitura

Lei de diretrizes e bases da educação nacional
(Seção III)

Art. 32. O ensino fundamental obrigatório [...] terá por objetivo a formação básica do cidadão, mediante:

I - o desenvolvimento da capacidade de aprender, tendo como meios básicos o pleno domínio da leitura, da escrita e do cálculo.

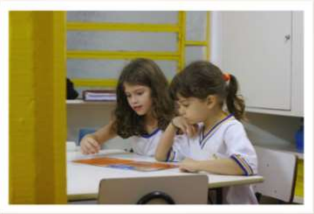

A importância da leitura

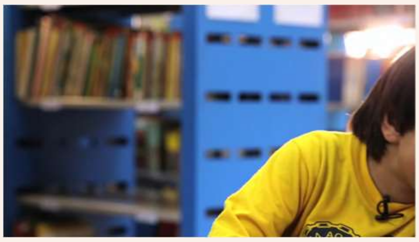

Antes da Pandemia de COVID-19...

- 54,73% dos alunos concluintes do 3º ano do EF apresentaram desempenho insuficiente no exame de proficiência em leitura nos resultados da Avaliação Nacional da Alfabetização (ANA, 2016).

  - Terminar o 3º ano do EF sem saber ler compromete a trajetória escolar da criança, refletindo em **altas taxas de reprovação, distorção idade-série, abandono e evasão (PNA).**

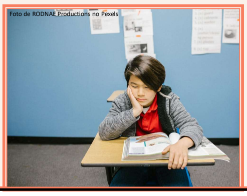
Foto de RODNAE Productions no Pexels

Na Pandemia de COVID-19...

- Zhao et al. (2020) identificaram diminuição do interesse dos alunos para desenvolver habilidades de aprendizagem.
- 70% das crianças com 10 anos de idade são incapazes de compreender um texto simples nos países de baixa e média renda (UNICEF).
- 80% das crianças ao final do ensino fundamental I são agora incapazes de compreender um texto simples, comparado a 50% antes da pandemia (UNICEF).

E agora?

A leitura na escola deve se adaptar para alcançar a interação entre o leitor do texto, o próprio texto e seu autor, os colegas da sala de aula e também com o professor.

O processo de leitura. Uma perspectiva interativa

Modelos hierárquicos ascendente *buttom up*

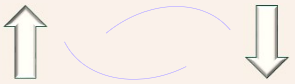

Modelos hierárquicos descendente *top down*

Para isso...

...é preciso ter noções de estratégias de leitura para os diferentes níveis de ensino e, o mais importante, o professor deve ser um leitor.

Para refletir: Qual o seu livro favorito? Por quê?

As estratégias de leitura são **um dos caminhos possíveis** para atingir uma formação do leitor proficiente.

O que são estratégias de leitura?

"...são procedimentos conscientes ou inconscientes utilizados pelo leitor para decodificar, compreender e interpretar o texto e resolver os problemas que encontra durante a leitura" (MENEGASSI, 2005, p.77 apud JAROSLAVA & PIANARO, 2013).

selecionar

antecipar

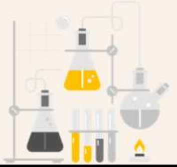

verificar

Para inspirar...

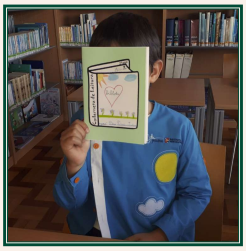

Esta Foto de Autor Desconhecido está licenciado em CC BY-NC-ND

Boas práticas

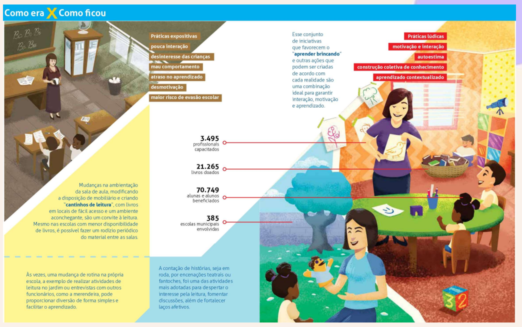

Retirado da cartilha "Programa Educação Integrada: boas práticas para a alfabetização de cada menina e menino"

(Unicef, 2019)

Programa de intervenção no 3º ano

Mafalda Ferreira e Carolina Gonçalves (2018)

Participantes

20 alunos (13 meninos e 7 meninas)

3 alunos com NEE

3 alunos em avaliação

Método

- Brochura com as estratégias de compreensão;
- Estratégias para antes, durante e após a leitura;
- Pré-teste, avaliação intermédia e pós-teste

Figura 1- Brochura com as estratégias de compreensão leitora (Ferreira; Gonçalves, 2018).

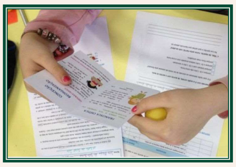

Programa de intervenção no 3º ano

Mafalda Ferreira e Carolina Gonçalves (2018)

Resultados

Tabela 1- Classificações dos alunos em percentagem nos momentos de avaliação do nível de compreensão leitora (Ferreira; Gonçalves, 2018).

	Pré-teste	Avaliação intermédia	Pós-teste
Fraco	0%	0%	0%
Não satisfaz	10%	9%	5%
Satisfaz	20%	38%	15%
Satisfaz bem	60%	29%	35%
Excelente	10%	24%	45%
Média	69	69.5	80

Figura 2-Gráfico de resultados relativos à média de percentagens para cada um dos parâmetros da compreensão leitora (Ferreira; Gonçalves, 2018).

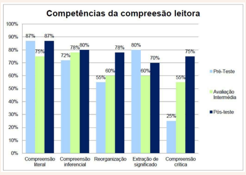

Estratégias de compreensão leitora

- Um processo para formar um leitor autônomo e ativo;
- Devem estar presentes ao longo de toda a atividade.

antes ⇦ ⇨ depois

Checklist de Acompanhamento Semanal

Quantas vezes você leu com os seus alunos na semana? *

- Em todas as aulas
- Em algumas aulas
- Não lemos na semana

Como você avalia a participação e o desempenho dos alunos na leitura? *

- Excelente
- Muito bom
- Bom
- Pouca participação/baixo desempenho

Gostaria de acrescentar alguma justificativa ou comentário? *

Sua resposta

Enviar

Referências

BRASIL. Lei no 9.394, de 20 de dezembro de 1996. *Estabelece as diretrizes e bases da educação nacional. República Federativa do Brasil.* Disponível em: http://www.planalto.gov.br/ccivil_03/leis/l9394.htm. Acesso em: 28 de agosto de 2022.

A Importância da Leitura. Disponível em: <https://www.youtube.com/watch?v=i8PYvikL2g8>. Acesso em: 28 ago. 2022.

SOLÉI. *Estratégias de leitura*. Porto Alegre, RS: Artmed, 1998.

BRASIL. *PNA Política Nacional de Alfabetização/Secretaria de Alfabetização*. Ministério da Educação. Secretaria de Alfabetização. Brasília: MEC, SEALF, 2019.

UNICEF. *70% das crianças com 10 anos de idade encontram-se agora em situação de pobreza de aprendizagem, incapazes de ler e compreender um texto simples*. Disponível em: <https://www.unicef.org/brazil/comunicados-de-imprensa/70-por-cento-das-criancas-com-10-anos-de-idade-encontram-se-agora-em-situacao-de-pobreza-de-aprendizagem>.

FERREIRA, M.; GONÇALVES, C. Do ensino explícito de estratégias de compreensão leitora ao sucesso na aprendizagem da leitura: programa de intervenção no 3.º ano de escolaridadeFrom the explicit teaching of reading comprehension strategies to the success in reading learning. *Saber & Educar*, n. 25, 31 dez. 2018. Acesso em: 13 abr. 2021.

UNICEF. *Programa Educação Integrada*. Disponível em: <https://www.unicef.org/brazil/relatorios/programa-educacao-integrada-boas-praticas>. Acesso em: 29 ago. 2022.

ZHAO, Y. *et al.* The Effects of Online Homeschooling on Children, Parents, and Teachers of Grades 1–9 During the COVID-19 Pandemic. *Medical Science Monitor: International Medical Journal of Experimental and Clinical Research*, v. 26, p. e925591-1e925591-10, 12 set. 2020. Disponível em: <https://www.ncbi.nlm.nih.gov/pmc/articles/PMC7507793/>.

Rayra Santos de Souza
Maria Clara Neder
Maria Cristina Triguero Veloz Teixeira

Universidade Presbiteriana Mackenzie

Obrigada!

Informações de contato:
rayrassouza07@gmail.com
(11) 9 9237-8267

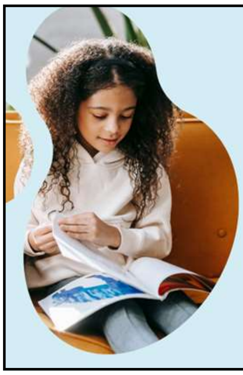

Aula 2

Tema de hoje:

Estratégias para antes da leitura

Estratégias para antes da leitura

Segundo Solé (1998), a divisão de estratégias para antes, durante e depois da leitura tem um caráter explicativo, mas elas são passíveis de troca na prática.

Pontos importantes:

Ideias gerais;
Motivação para a leitura;
Objetivos da leitura;
Revisão e atualização do conhecimento prévio;
Estabelecimento de previsões sobre o texto;
Formulação de perguntas.

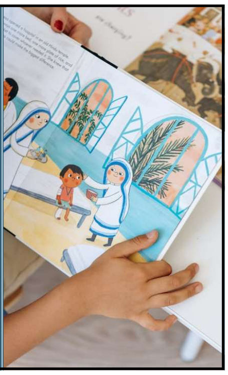

Ideias gerais

Atividade prazerosa: crianças e professores motivados para aprender e ensinar a ler;

Ler por ler X trabalhar a leitura: o exemplo do professor;

Leitura como competição: pode gerar sentimentos de incompetência e contribuir para o fracasso do aluno que apresenta dificuldades;

Complexidade e capacidade: ajudar a superar desafios.

Motivação para a leitura

- ✓ A criança tem de saber o que deve fazer;
- ✓ Sentir que é capaz de fazê-lo;
- ✓ Achar interessante o que se propõe que ela faça.

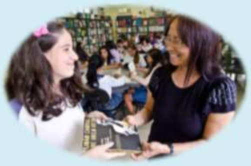

Objetivos da leitura

Objetivos da leitura que são importantes na vida adulta e podem ser trabalhados na escola:

Ler para obter uma informação;
Ler para seguir instruções;
Ler para aprender;
Ler para revisar um escrito próprio;
Ler por prazer;
Ler para comunicar algo;
Ler para praticar a leitura em voz alta;
Ler para verificar o que se compreendeu.

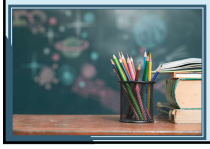

Revisão e atualização do conhecimento prévio

1. Dar alguma explicação geral sobre o que será lido;
2. Ajudar os alunos a prestar atenção a determinados aspectos do texto que podem ativar seu conhecimento prévio;
3. Incentivar os alunos a exporem o que já sabem sobre o tema.

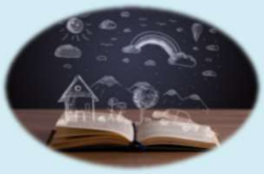

Estabelecimento de previsões

Formular hipóteses, fazer previsões, exige correr riscos;

Previsões que podem ser feitas antes da leitura:

TÍTULOS – ILUSTRAÇÕES – EXPERIÊNCIAS

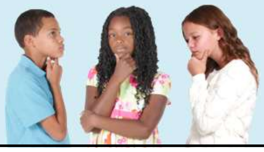

Formulação de perguntas

A partir das previsões pode ser útil formular perguntas concretas a que se gostaria de responder mediante a leitura.

"com a leitura vão ver se é verdade tudo o que disseram" X *"leiam a página 36"*

Estratégias para antes da leitura

O que pode ser feito antes da leitura tem a finalidade de:

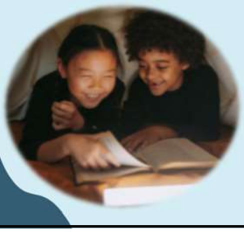

- ✓ Suscitar a necessidade de ler;
- ✓ Transformar o aluno em um leitor ativo.

Referências

SOLÉI. *Estratégias de leitura*. Porto Alegre, RS: Artmed, 1998.

TARAS, Jaroslava; ANGELO, Cristiane Malinoski Pianaro. *Estratégias de leitura para a formação e o desenvolvimento do aluno-leitor da sala de apoio à aprendizagem de língua portuguesa.* In: PARANÁ. Secretaria de Estado da Educação. Superintendência de Educação. Os Desafios da Escola Pública Paranaense na Perspectiva do Professor PDE, 2013. Curitiba: SEED/PR., 2016. V.1. (Cadernos PDE). Disponível em: <http://www.gestaoescolar.diaadia.pr.gov.br/modules/conteudo/conteudo.php?conteudo=20>. Acesso em 01/09/2022. ISBN 978-85-8015-076-6.

Rayra Santos de Souza
Maria Clara Neder
Maria Cristina Triguero Veloz Teixeira

Universidade Presbiteriana Mackenzie

Obrigada!

Informações de contato:
rayrassouza07@gmail.com
(11) 9 9237-8267

Aula 3

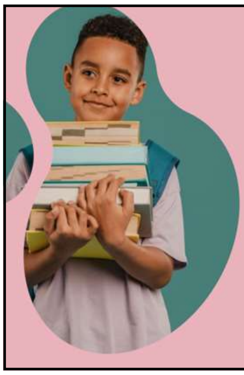

Tema de hoje:

Estratégias de compreensão para a leitura

O processo de leitura

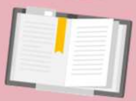

A leitura é um processo de **emissão** e **verificação** de previsões que levam à construção da compreensão do texto.

O processo de leitura deve garantir que o leitor compreenda diversos textos.

O processo de leitura

Não existe uma determinada sequência temporal: as etapas às quais nos referimos sucedem em diferentes situações de leitura de complexidade diversa.

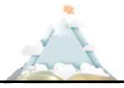

O processo de leitura

Alunos e alunas **sempre** podem aprender a ler melhor mediante as intervenções do professor.

Ler o nome de um colega X o título de uma história X textos complexos.

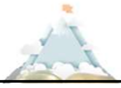

A leitura compartilhada

A leitura compartilhada permite que os alunos compreendam e usem as estratégias úteis para compreender os textos.

- Formular previsões sobre o texto;
- Formular perguntas sobre o que foi lido;
- Esclarecer possíveis dúvidas;
- Resumir as ideias do texto.

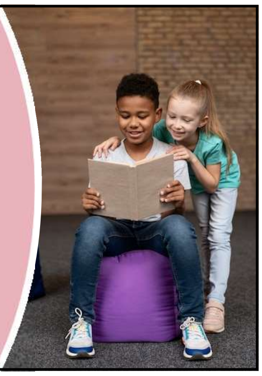

Tarefas de leitura compartilhada

A divisão da responsabilidade de organizar a tarefa de leitura e de envolver os outros.

Quatro estratégias básicas para o moderador/responsável:

- Fazer um resumo do que foi lido para o grupo e solicitar sua concordância;
- Pedir explicações ou esclarecimentos sobre determinadas dúvidas do texto;
- Formular uma ou algumas perguntas às crianças, cuja resposta torna a leitura necessária;
- Estabelecer previsões sobre o que ainda não foi lido.

Ciclo: ler, resumir, solicitar esclarecimentos e prever.

Tarefas de leitura compartilhada

Possibilidades de variação:

- Trabalho com pequenos grupos;
- Variações na sequência (perguntar, esclarecer, recapitular, prever);
- Formular perguntas com os alunos;

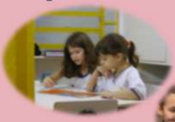

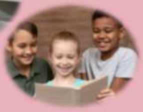

Resumindo...

As atividades de leitura compartilhada devem permitir a transferência da responsabilidade das mãos do professor para as mãos do aluno.

O papel do professor na leitura compartilhada

- ✓ Avaliar o que pode ou não pedir aos alunos e o tipo de ajuda de que estes vão precisar;
- ✓ Planejar a tarefa de leitura;
- ✓ Observar os alunos para oferecer desafios e apoios.

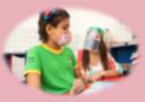

Quando a leitura compartilhada pode ser usada?

Desde os níveis iniciais para que os alunos possam assumir um papel ativo na própria leitura e aprendizagem.

A leitura independente

A escola pode se propor o objetivo de promover o uso de determinadas estratégias em tarefas de leitura individual.

- Inserir perguntas e lembretes no meio do texto;
- Colocar erros no texto e solicitar que os alunos encontrem;
- Deixar lacunas para serem completadas.

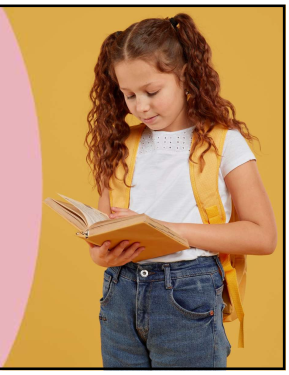

Os resultados na prática...

Os resultados na prática...

Intervenção baseada em leitura compartilhada de histórias: efeito nas tarefas de baixo e alto nível de leitura e escrita (Zuanett; Novaes; & Fukuda, 2020).

Método: 44 crianças divididas em dois grupos: G1 (crianças com dificuldade em compreensão leitora) e G2 (sem dificuldades).

Programa de intervenção: 15 encontros que estimulava a leitura compartilhada de histórias.

Quadro 1. Breve descrição de cada sessão do programa de intervenção com base na leitura compartilhada de histórias utilizado neste estudo.

1ª etapa	Antes da leitura	Introdução da história: descrição do cenário e personagens
	Durante a leitura	Compreensão dirigida (ex: qual o nome do personagem?)
	Após a leitura	A criança desenhava a parte que mais gostou da história
2ª etapa	Antes da leitura	Introdução expandida: análise detalhada. Localização da ideia central, relembrando detalhes da história, esclarecendo dúvidas do último encontro
	Durante a leitura	Automonitoramento da compreensão (ex: qual a ideia deste paragráfo?)
	Após a leitura	A criança escrevia três acontecimentos da história, em adequada sequência temporal
3ª etapa	Antes da leitura	Leitura direcionada: exploração das figuras do livro a depender da pergunta da pesquisadora
	Durante a leitura	Antes de responder as perguntas, cada criança tinha um minuto para pensar na resposta e formular a resposta. Sua resposta deveria ter detalhes e um vocabulário diferente do que foi lido
	Após a leitura	Entregue figuras e frases relacionadas a história. Cada criança deveria organizar essas em ordem
4ª etapa	Antes da leitura	Relembrando o livro: Questionamento a respeito de detalhes da história
	Durante a leitura	História com clozes: preencher as lacunas durante a leitura de cada paragráfo e revisão da atividade
	Após a leitura	Cada criança deveria mudar algum acontecimento da história, escrevendo um novo final
5ª etapa	Antes da leitura	Auto questionamento: cada criança deveria fazer perguntas a si mesmo e anotar no caderno
	Durante a leitura	História com clozes: preencher as lacunas durante a leitura, porém a palavra colocada deveria ser sinônimo da que está no texto
	Após a leitura	Mudança das características do personagem e promovendo um novo desfecho

Os resultados na prática...

Intervenção baseada em leitura compartilhada de histórias: efeito nas tarefas de baixo e alto nível de leitura e escrita (Zuanett; Novaes; & Fukuda, 2020).

Resultados:
Após a intervenção as crianças do G1 apresentaram melhora significativa nas variáveis avaliadas.

Conclusão: A leitura compartilhada promoveu o desenvolvimento das habilidades de baixo e alto nível de leitura e escrita.

Referências

MITSUNARI, P. *Efeitos de um programa de leitura compartilhada em contexto familiar sobre habilidades de leitura e padrões emocionais e comportamentais de crianças*. 2019. Dissertação de Mestrado – Programa de Pós-Graduação em Distúrbios do Desenvolvimento da Universidade Presbiteriana Mackenzie, 2019.

SOLÉI. *Estratégias de leitura*. Porto Alegre, RS: Artmed, 1998.

ZUANETTI, P. A.; NOVAES, C. B.; FUKUDA, M. T. H. Intervenção baseada em leitura compartilhada de histórias: efeito nas tarefas de baixo e alto nível de leitura e escrita. *CoDAS*, v. 33, n. 3, 2020. Acesso em: 2 fev. 2022.

Rayra Santos de Souza
Maria Clara Neder
Maria Cristina Triguero Veloz Teixeira

Universidade Presbiteriana Mackenzie

Obrigada!

Informações de contato:
rayrassouza07@gmail.com
(11) 9 9237-8267

Aula 4

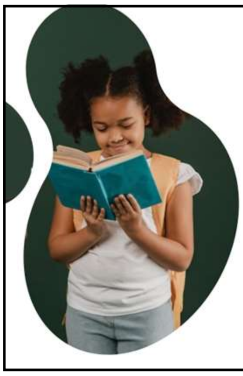

Tema de hoje:

Estratégias para depois da leitura

Compreendendo e aprendendo

Identificação da ideia principal, elaboração de resumo e formulação e resposta de perguntas.

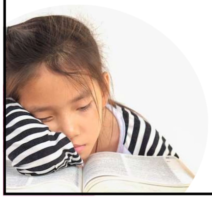

Sobrecarga de informação

↓

Desmotivação na leitura

Compreendendo e aprendendo

Houve confirmação das hipóteses?

Objetivo: Identificar tema e ideia principal do texto; elaborar resumo das ideias principais e secundárias.

Mais geral → Mais preciso

A ideia geral

Objetivos de leitura + conhecimentos prévios + informação transmitida no texto

"O mais importante deste capítulo?"

"O que o autor quis transmitir?"

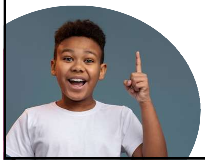

O que é a ideia geral? Para que serve?
Como ela é identificada e gerada?

A ideia geral

- ✓ Explicar e exemplificar;
- ✓ Discutir o processo da leitura;
- ✓ Propor tarefa compartilhada;
- ✓ Priorizar textos com ideia principal no início;
- ✓ Possibilitar discussão conjunta.

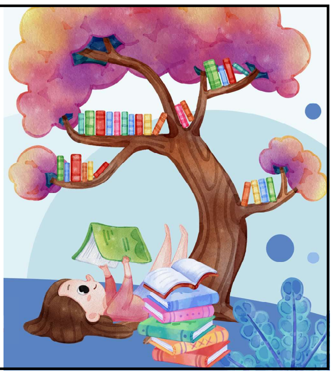

Resumo

Tema + ideias gerais + ideias secundárias

→ Produção escrita

- ✓ Meio para compreender e aprender;
- ✓ Tarefa complexa, mas pode ser estimulada desde o início da alfabetização (relato oral).

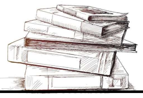

Propostas para se inspirar

Propostas para se inspirar

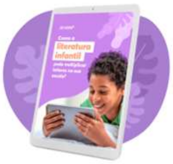

Projeto Árvore

Como a literatura infantil pode multiplicar leitores na sua escola?

Dicas para levantar o debate sobre literatura infantil entre os professores;

Neste material, preparado para educadores, você encontrará dicas para levantar o debate sobre literatura infantil entre os professores e também sugestões de livros.

Propostas para se inspirar

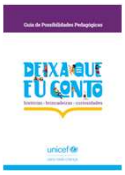

Iniciativa do UNICEF (2021) com o objetivo de levar histórias, brincadeiras e curiosidades por meio de programas em áudio de 30 minutos.

Neste Guia de Possibilidades Pedagógicas, são apresentadas propostas que podem inspirar educadores e famílias, e contribuir de maneira ativa e criativa para a utilização dos *podcasts* com as crianças.

Considerações Finais

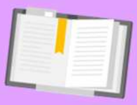

- ✓ Ensinar a formular perguntas leitor → autônomo e ativo;
- ✓ Aprender a ler significa aprender a encontrar sentido e interesse na leitura;
- ✓ Aprender a ler requer que se ensine a ler (ensinar a fazer além de orientar);
- ✓ A leitura deve ser abordada como uma questão de equipe nas diferentes etapas, graus de ensino e nas escolas.
